# Lef1-dependent hypothalamic neurogenesis inhibits anxiety

**DOI:** 10.1371/journal.pbio.2002257

**Published:** 2017-08-24

**Authors:** Yuanyuan Xie, Dan Kaufmann, Matthew J. Moulton, Samin Panahi, John A. Gaynes, Harrison N. Watters, Dingxi Zhou, Hai-Hui Xue, Camille M. Fung, Edward M. Levine, Anthea Letsou, K. C. Brennan, Richard I. Dorsky

**Affiliations:** 1 Department of Neurobiology and Anatomy, University of Utah, Salt Lake City, Utah, United States of America; 2 Department of Neurology, University of Utah, Salt Lake City, Utah, United States of America; 3 Department of Human Genetics, University of Utah, Salt Lake City, Utah, United States of America; 4 School of Life Sciences, Peking University, Beijing, China; 5 Department of Microbiology, Carver College of Medicine, University of Iowa, Iowa City, Iowa, United States of America; 6 Division of Neonatology, Department of Pediatrics, University of Utah School of Medicine, Salt Lake City, Utah, United States of America; 7 Department of Ophthalmology and Visual Sciences, John A. Moran Eye Center, University of Utah, Salt Lake City, Utah, United States of America; Yale School of Medicine, United States of America

## Abstract

While innate behaviors are conserved throughout the animal kingdom, it is unknown whether common signaling pathways regulate the development of neuronal populations mediating these behaviors in diverse organisms. Here, we demonstrate that the Wnt/ß-catenin effector Lef1 is required for the differentiation of anxiolytic hypothalamic neurons in zebrafish and mice, although the identity of Lef1-dependent genes and neurons differ between these 2 species. We further show that zebrafish and *Drosophila* have common Lef1-dependent gene expression in their respective neuroendocrine organs, consistent with a conserved pathway that has diverged in the mouse. Finally, orthologs of Lef1-dependent genes from both zebrafish and mouse show highly correlated hypothalamic expression in marmosets and humans, suggesting co-regulation of 2 parallel anxiolytic pathways in primates. These findings demonstrate that during evolution, a transcription factor can act through multiple mechanisms to generate a common behavioral output, and that Lef1 regulates circuit development that is fundamentally important for mediating anxiety in a wide variety of animal species.

## Author summary

Humans, mice, fish, and even flies exhibit anxiety-like behavior despite the fact that their brain anatomy varies widely. This study reveals another common thread that runs through these diverse animals: the molecular origins of their shared behavior. Gene knockout experiments in mouse and zebrafish show that the molecular signal Wnt acts through the transcription factor Lef1 to inhibit anxiety in both species. The pathway is required for formation of anxiolytic neurons in a highly conserved brain region, the hypothalamus. From there, however, the process diverges. In the fish, the pathway triggers genes including *corticotropin-releasing hormone binding protein* (*crhbp*), but in mice the same pathway calls into action a different gene, *Pro-melanin concentrating hormone* (*Pmch*). By comparison, the fruit fly *Drosophila* activates *crhbp*, similar to zebrafish. Furthermore, *CRHBP* and *PMCH* show extraordinarily coordinated expression in the primate hypothalamus, indicating that they may act together downstream of Wnt and Lef1 to regulate human behavior. This work reveals the surprising finding that conserved signaling pathways can regulate common behavioral outputs through diverse brain circuits during evolution.

## Introduction

Recent work has demonstrated that innate behaviors can be highly conserved across diverse animal models [1]. Individual neuronal populations that mediate these behaviors are specified during embryogenesis by transcription factors that can also be conserved across species [2]. However, molecular signaling pathways that regulate the development of common behavioral circuits have not been identified. As brain anatomy and connectivity change through evolution, it is possible that a single pathway could act through diverse molecular and cellular targets to establish a single behavioral output, which is the ultimate constraint on gene function.

Wnt/ß-catenin signaling plays important evolutionarily conserved roles in brain development, and thus represents an ideal candidate pathway to link gene regulation with the evolution of behavioral circuits. The Wnt pathway acts through Tcf/Lef transcription factors [3], and both Wnt signaling and Lef1 are required for neurogenesis in the zebrafish hypothalamus [4], an evolutionarily ancient brain structure that regulates innate behaviors [5]. However, the identity and behavioral function of Lef1-dependent hypothalamic neurons, and their degree of evolutionary conservation, are unknown. Here, we show that Lef1 is required for the differentiation of hypothalamic neurons that inhibit anxiety in both zebrafish and mice, but through divergent molecular and cellular mechanisms in the 2 species. Generation of neurons expressing *corticotropin-releasing hormone binding protein* (*crhbp*) requires Lef1 in zebrafish but not in mice, whereas neurons expressing *Pro-melanin concentrating hormone* (*Pmch*) are Lef1-dependent in mice but not in zebrafish. Furthermore, zebrafish and *Drosophila* have common Lef1-dependent *crhbp* expression in their respective neuroendocrine organs, consistent with an ancient conserved pathway that has diverged in mammals. Finally, the Genotype-Tissue Expression (GTEx) project [6] reveals a top-ranked positive correlation between *CRHBP* and *PMCH* in the human hypothalamus, suggesting co-expression and/or co-regulation. Both genes are also correlated with *LEF1* expression in humans, and are expressed in the same region of the marmoset hypothalamus, consistent with a conserved regulatory pathway in primates. These findings suggest that the gene expression network regulated by a transcription factor can change during evolution while still generating a common behavioral output. Our data also suggest an anxiolytic role for Wnt signaling in the human hypothalamus, with potential implications for the etiology and treatment of anxiety disorders.

## Results

### Lef1 is required for the differentiation of hypothalamic neurons in zebrafish

**Fig 1:**
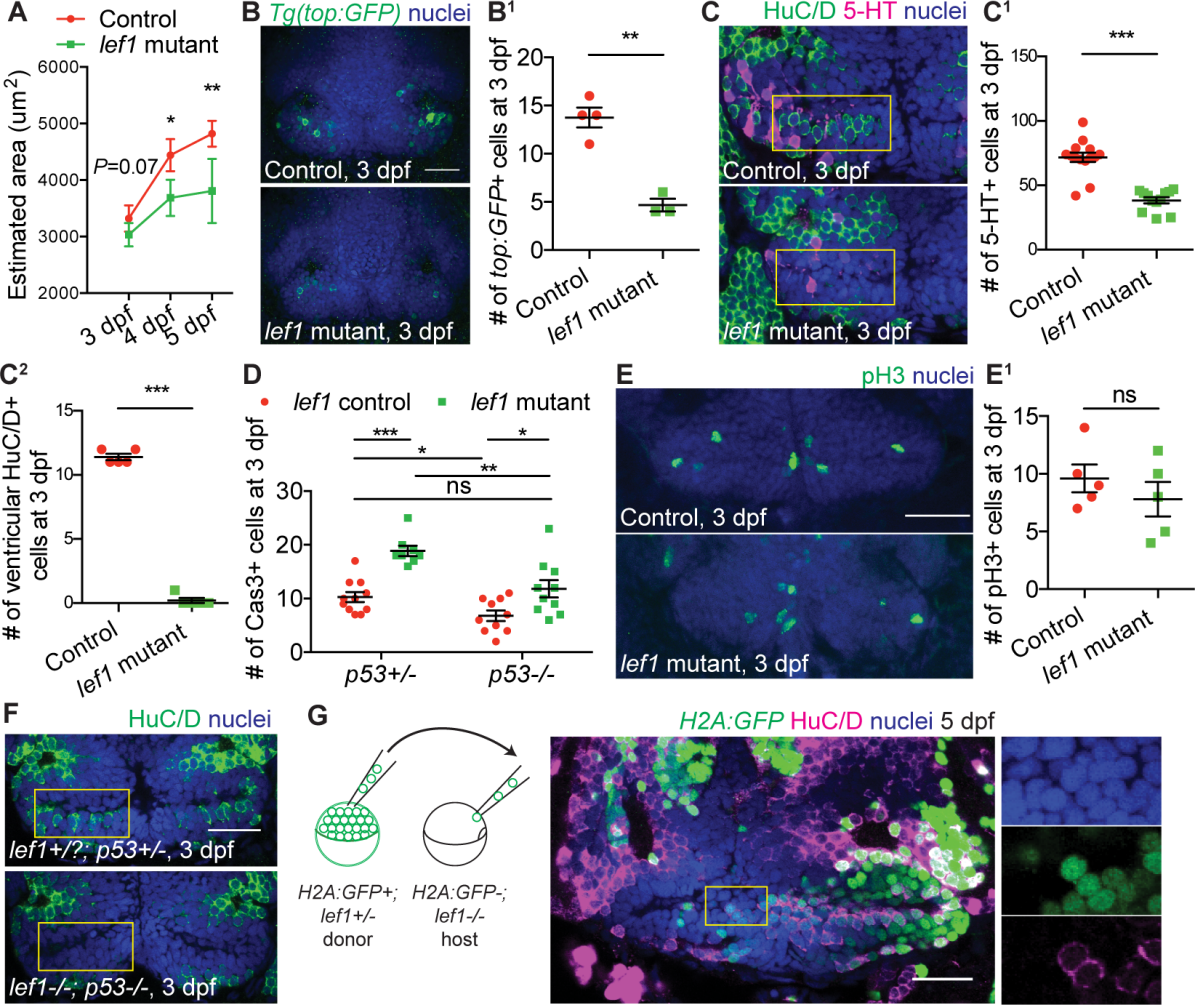
Lef1 promotes neurogenesis in the zebrafish caudal hypothalamus (Hc). (A) Estimation of Hc size in control and *lef1* mutants. See S1B Fig for method. (B-F) Immunostaining and quantification in 3 days post-fertilization (dpf) Hc. Representative immunostaining images of Wnt-responsive *Tg(top*:*GFP)+* (B), 5-HT+ and HuC/D+ (C), and mitotic phospho-histone H3-positive (pH3+) cells (E) in control and *lef1* mutants are shown on the left and quantified on the right (B^1^, C^1^, C^2^ and E^1^). Quantification of apoptotic active Caspase3+ (Cas3+) cells on the *p53* mutant background is shown in (D), and representative immunostaining images of HuC/D+ cells are shown in (F). (G) Transplantation (schematic on the left) followed by HuC/D immunostaining at 5 dpf. All yellow rectangles depict the region with ventricular HuC/D+ cells normally present in wild-type (wt) fish, and magnified images in (G). All images show ventral views of whole-mounted brain with anterior on top. Data are mean ± SEM, except mean ± SD in (A). ***P < 0.001, **P < 0.01, *P < 0.05, ns. *P* > 0.05 by unpaired Student *t* tests. Scale bars: 25 μm. See S1 Table for description of confocal imaging, quantification and experimental *n*. Raw data can be found in S1 Data.

We sought to first characterize the earliest cellular defect in *lef1* null zebrafish mutants [4], so that we could perform a transcriptome analysis at that stage to identify Lef1-dependent genes. Despite grossly normal morphology, mass, and brain size, *lef1* mutants have a smaller caudal hypothalamus (Hc) at 15 days post-fertilization (dpf) [4], and we found that the size reduction occurred at as early as 3–4 dpf (Fig 1A and S1A and S1B Fig). At 3 dpf the tissue already contained fewer Wnt-responsive cells [7] (Fig 1B), as well as fewer serotonergic cells and ventricular GABAergic HuC/D+ neurons (Fig 1C and S1C Fig). However, *th2*:*GFP*+ dopaminergic neurons [8] were unaffected (S1D Fig), indicating that not all neuronal subtypes are Lef1-dependent. In addition, the number of BLBP+ cells was increased (S1E Fig), confirming an inhibitory role of Wnt signaling in the formation of hypothalamic radial glia [4,9].

To determine the cellular mechanism underlying the decreased populations in *lef1* mutants, we measured apoptosis and proliferation. We observed an increase in *p53*-dependent apoptosis within the Hc at 3 dpf (Fig 1D), but no change in proliferation at 3 dpf and beyond (Fig 1E and S1F–S1H Fig). Rescue of apoptosis by loss of *p53* (Fig 1D) did not restore HuC/D expression in *lef1* mutants (Fig 1F), consistent with a primary defect in progenitor differentiation. To confirm a failure in neurogenesis, we performed BrdU pulse-chase experiments, and observed fewer newly born serotonergic and ventricular HuC/D+ cells in *lef1* mutants (S1I Fig). To test whether Lef1 functions cell-autonomously, we transplanted cells from *lef1+/-* donors into the hypothalamic anlage of *lef1* mutant hosts during gastrulation, and observed rescue of ventricular HuC/D expression only in donor cells (Fig 1G). Together these data suggest that Lef1 functions cell-autonomously to promote hypothalamic neurogenesis; in *lef1* mutants, neural progenitors fail to differentiate and subsequently undergo cell death, leading to a smaller Hc. Our data also justified 3 dpf as the optimal time point to perform a transcriptome analysis.

### Lef1-dependent genes in the zebrafish hypothalamus are associated with anxiety

**Fig 2:**
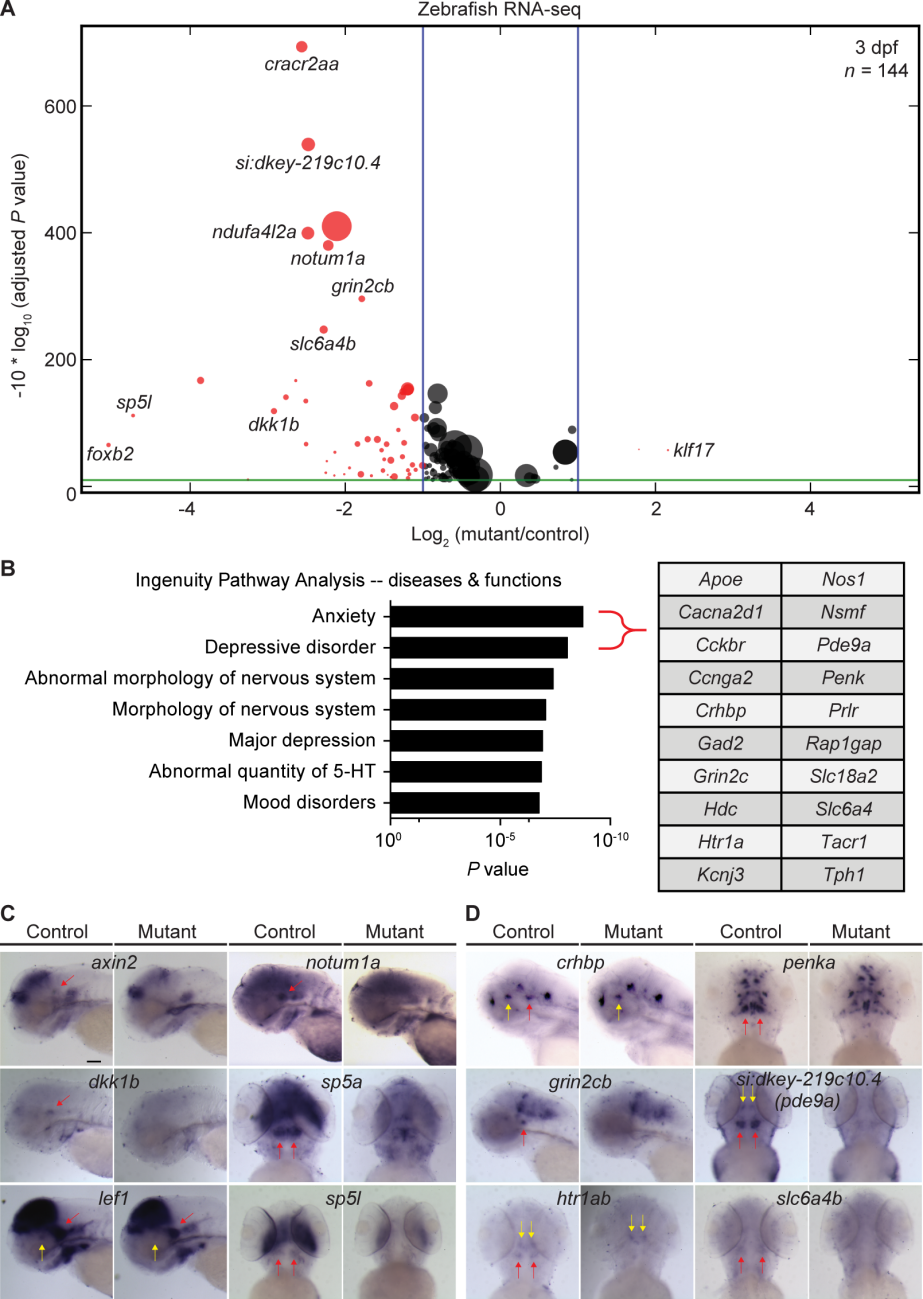
Lef1 activates expression of zebrafish hypothalamic genes associated with anxiety. (A) Volcano plot of zebrafish RNA sequencing (RNA-seq) shows differentially expressed genes in the 3 days post-fertilization (dpf) hypothalamus of *lef1* mutants compared to control. Only genes with adjusted *P* value (AdjP) <0.1 (green line) are shown. Genes with an absolute value of log_2_ ratio >1 (blue lines) are shown in red; others are shown in black. Node size represents the averaged fragments per kilobase of transcript per million mapped reads of a gene in the control. (B) Ingenuity Pathway Analysis (IPA) for zebrafish hypothalamic Lef1*-*dependent genes revealed 20 genes associated with anxiety and depressive disorder, listed in the table. (C and D) Representative images of whole mount in situ hybridization on 3 dpf control and *lef1* mutant embryos for known Wnt targets (C) and genes associated with anxiety and depressive disorder (D). Red and yellow arrows indicate expression in caudal and rostral hypothalamus, respectively. Lateral (*axin2*, *dkk1b*, *lef1*, *notum1a*, *crhbp*, and *grin2cb*) or ventral (other genes) views were selected for optimal expression visualization. Scale bar: 100 μm.

To identify Lef1-dependent genes, we next performed RNA sequencing (RNA-seq) analysis of whole hypothalami dissected from 3 dpf control and *lef1* mutant zebrafish embryos, and found 144 genes with an adjusted *P* value (AdjP) <0.1, among which 53 genes had a fold change >2 (Fig 2A, S2 Table). Most of these genes had reduced expression in *lef1* mutants (Fig 2A), consistent with Lef1 functioning as a Wnt transcriptional activator [10]. Surprisingly, Ingenuity Pathway Analysis (IPA) identified Lef1-dependent genes as being most highly associated with anxiety and depressive disorder (Fig 2B and S3 and S4 Tables). In contrast, genes associated with other hypothalamus-mediated behaviors, such as feeding (*neuropeptide Y* [*npy*], *agouti-related protein* [*agrp*], and *proopiomelanocortin* [*pomc*]) or sleep (*hypocretin* [*hcrt*]), were unaffected (S2 Table). We performed in situ hybridization on 3 dpf offspring of *lef1+/-* incrosses and confirmed that all Lef1-dependent genes with specific detectable hypothalamic expression showed predicted changes in approximately 25% of embryos, consistent with Mendelian segregation (Fig 2C and 2D and S2A–S2C Fig). These included several known Wnt targets such as *sp5a* and *sp5l* [11] (Fig 2C), and anxiety-related genes identified from IPA (Fig 2B and 2D). Expression of neuronal markers such as *crhbp* and *5-hydroxytryptamine receptor 1A b* (*htr1ab*), was lost specifically in the Hc of *lef1* mutants while remaining intact in the rostral hypothalamus (Fig 2D), resulting in their relatively small fold change in whole hypothalamus RNA-seq analysis (S2 Table). In contrast, expression of other genes, such as 2 *phosphodiesterase 9a* (*pde9a*) paralogs, was lost in the rostral hypothalamus and Hc of *lef1* mutants (Fig 2D and S2A Fig), consistent with *lef1* expression in both regions (Fig 2C). We also observed expression of Lef1-dependent genes in the Hc of wild-type (wt) adult zebrafish (S2D Fig), suggesting the presence of Wnt activity and Lef1-dependent neuronal populations throughout life. Together these results suggested that *lef1* mutants might have an anxiety-related behavioral phenotype.

### Zebrafish *lef1* mutants exhibit increased anxiety

**Fig 3:**
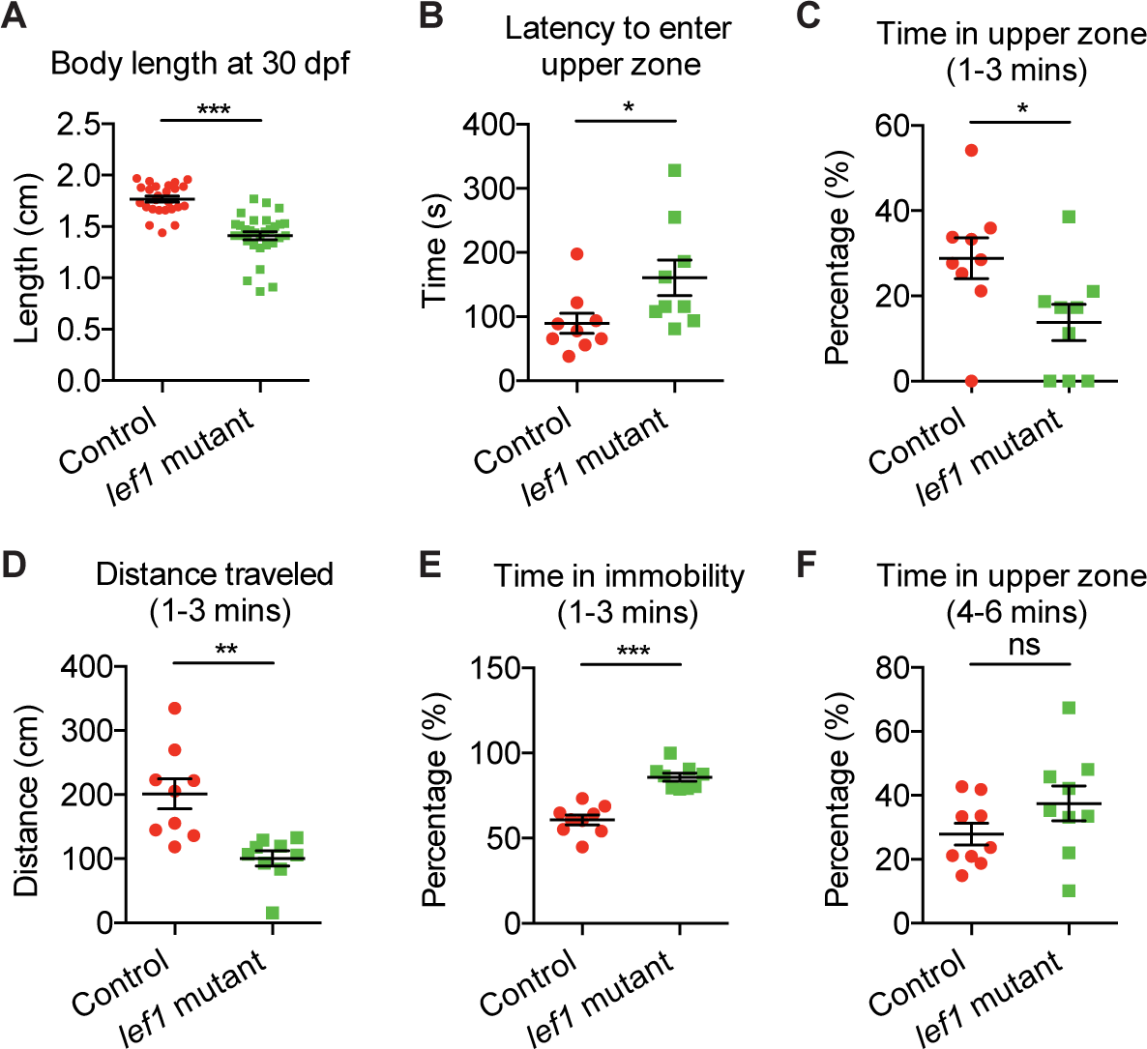
Lef1 regulates growth and anxiety in zebrafish. (A) Size of 30 days post-fertilization (dpf) fish when raised at 5 fish per tank separated by genotype. *n* = 25, 30 for control and mutant, respectively. (B-F) Novel tank diving test. Sixteen dpf larvae were analyzed between 1–3 minutes (C-E) or 4–6 minutes (F) after entering a novel tank. *n* = 9 for both controls and mutants. Data are mean ± SEM. *P < 0.05, **P < 0.01, ***P < 0.001, ns. *P* > 0.05 by unpaired Student *t* tests. Raw data can be found in S1 Data.

*lef1* mutants raised with siblings had decreased survival and size (S3A and S3C Fig). When separated at 15 dpf, mutants survived normally (S3B and S3C Fig), but were still smaller than control siblings at culture densities that maximized their growth (Fig 3A and S3D Fig), a phenotype potentially due to enhanced anxiety [12]. We then performed a novel tank diving test to measure anxiety-related behavior [13]. We found that *lef1* mutant larvae had a longer latency to enter the upper half of a novel tank and spent less overall time in this zone during the initial exploration phase (Fig 3B and 3C and S1 Video), consistent with elevated anxiety. Notably, *lef1* mutants travelled less distance during this phase, partially due to more frequent freezing behavior as indicated by increased time in immobility (Fig 3D and 3E and S1 Video), and again consistent with elevated anxiety. Importantly, *lef1* mutants no longer displayed anxiety-related behavior after the exploration phase (Fig 3F). The body growth and anxiety phenotypes in *lef1* mutants could be explained by reduced expression of multiple hypothalamic genes including *crhbp* (Fig 2D), which encodes a corticotropin-releasing hormone (CRH) inhibitor [14]. However, pleiotropic phenotypes in zebrafish *lef1* mutants [4,15] could also contribute to defects in growth or motor behavior. Therefore, we sought to create a tissue-specific mouse knockout model to examine the hypothalamic function of Lef1, and to determine whether it is evolutionarily conserved.

### Hypothalamic *Lef1* inhibits anxiety in mice

**Fig 4:**
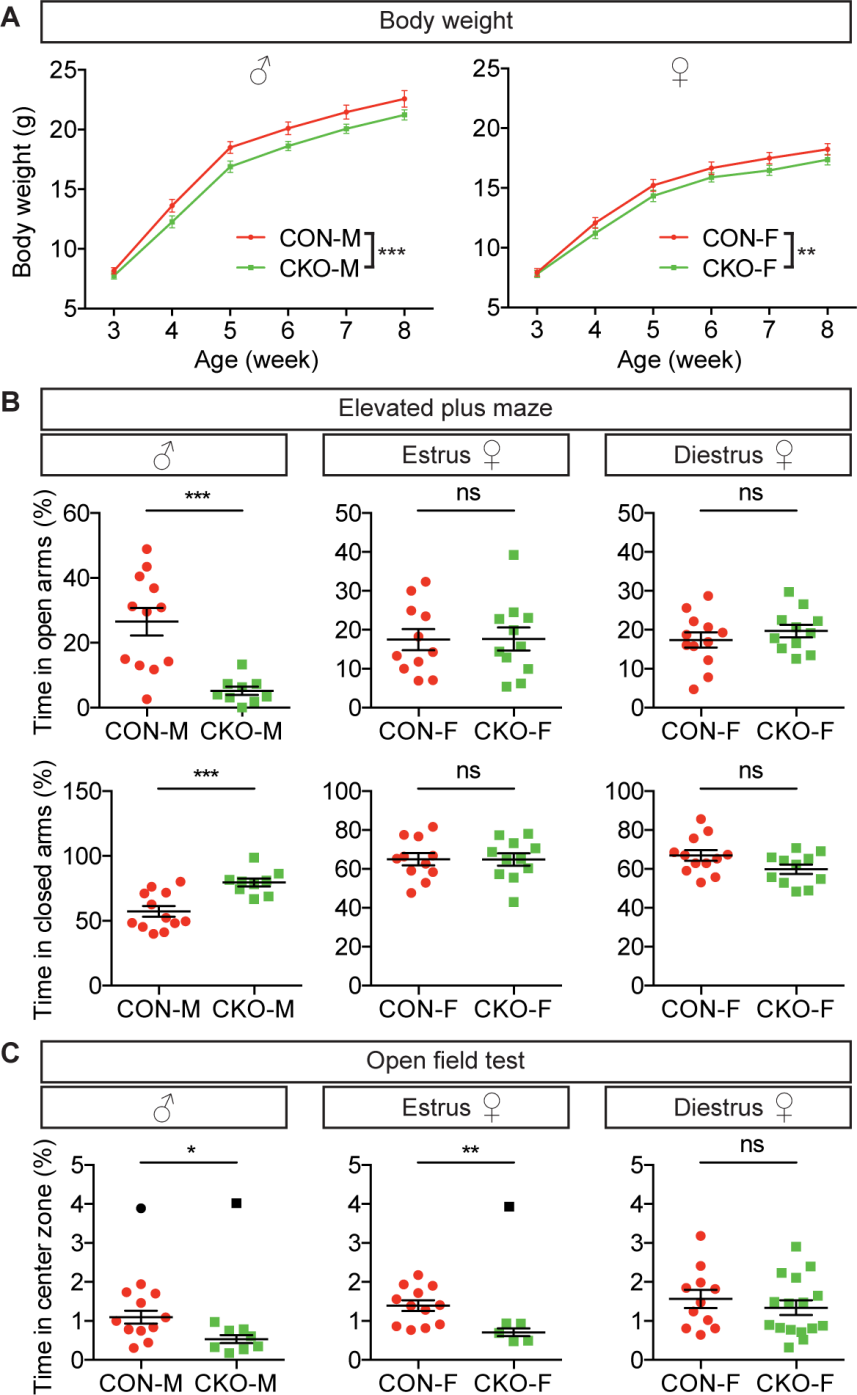
Hypothalamic Lef1 regulates growth and anxiety in mice. (A) Body weight of male *Lef1*^*CKO*^ (CKO-M, *n* = 27) and female *Lef1*^*CKO*^ (CKO-F, *n* = 26) compared to controls (CON-M, *n* = 27; CON-F, *n* = 26). (B) Elevated plus maze (EPM). (C) Open field test (OFT). In (B) and (C), *n* = 12, 9 for male CON, CKO. In (B), *n* = 11, 11 for female CON, CKO in estrus; *n* = 12, 11 for female CON, CKO in diestrus. In (C), *n* = 12, 6 for female CON, CKO in estrus; *n* = 11, 16 for female CON, CKO in diestrus. Data are mean ± 95% CI (A) or SEM (B and C). ***P < 0.001, **P < 0.01, *P < 0.05, ns. *P* > 0.05 by 2-way ANOVA with repeated measures (A, F_(1, 26)_ = 22.2 for male and F_(1, 25)_ = 8.842 for female) and unpaired Student *t* tests (B and C). Outliers depicted in black (C) were excluded using the Grubbs’ test (*P* < 0.05). Raw data can be found in S1 Data.

*Lef1* is expressed in the mouse Hc from embryonic day (E) 10.5 to adulthood [16,17], and while previously characterized *Lef1* null mutants exhibit postnatal lethality and a smaller body size, no hypothalamic phenotypes were reported [18,19]. We created a mouse hypothalamus knockout model using *Nkx2-1*^*Cre*^ and *Lef1*^*flox*^ alleles [20,21]. We also introduced the Cre reporter *Rosa*^*tdTomato*^ [22] to create the conditional knockout allele *Nkx2-1*^*Cre/+*^*;Lef1*^*flox/flox*^*;Rosa*^*tdTomato/+*^ (herein referred to as *Lef1*^*CKO*^) and control littermates *Nkx2-1*^*Cre/+*^*;Lef1*^*flox/+*^*;Rosa*^*tdTomato/+*^ (herein referred to as *Lef1*^*CON*^), which were used for all experiments. We confirmed successful recombination by tdTomato expression (S5A Fig), and loss of hypothalamic Lef1 and Wnt reporter [23] expression in *Lef1*^*CKO*^ mice (S5B and S5C Fig), which were viable, fertile, and morphologically indistinguishable from *Lef1*^*CON*^ littermates. However, both male and female *Lef1*^*CKO*^ mice gained weight more slowly after weaning (Fig 4A), similar to the phenotype we observed in zebrafish *lef1* mutants (Fig 3A), and again consistent with elevated anxiety [12].

To directly measure anxiety-related behavior, we used an elevated plus maze (EPM) test and found that male *Lef1*^*CKO*^ mice spent significantly less time in the open arms and more time in the closed arms (Fig 4B) despite normal mobility (S4A Fig). In an open field test (OFT), male *Lef1*^*CKO*^ mice spent significantly less time in the center zone (Fig 4C) despite normal mobility (S4B Fig). These results are consistent with elevated anxiety in male *Lef1*^*CKO*^ mice. We also observed enhanced anxiety specifically in OFT with estrous female *Lef1*^*CKO*^ mice, but not with diestrous or all females, or with EPM testing of any females (Fig 4B and 4C and S4A and S4B Fig), likely due to reported variations in anxiety-related behavior between different sexes [24] and different behavioral assays [25]. Together, these results suggest a conserved role of hypothalamic Lef1 in inhibiting anxiety.

### Hypothalamic *Lef1* is required for generation of *Pmch+* neurons in mice

**Fig 5:**
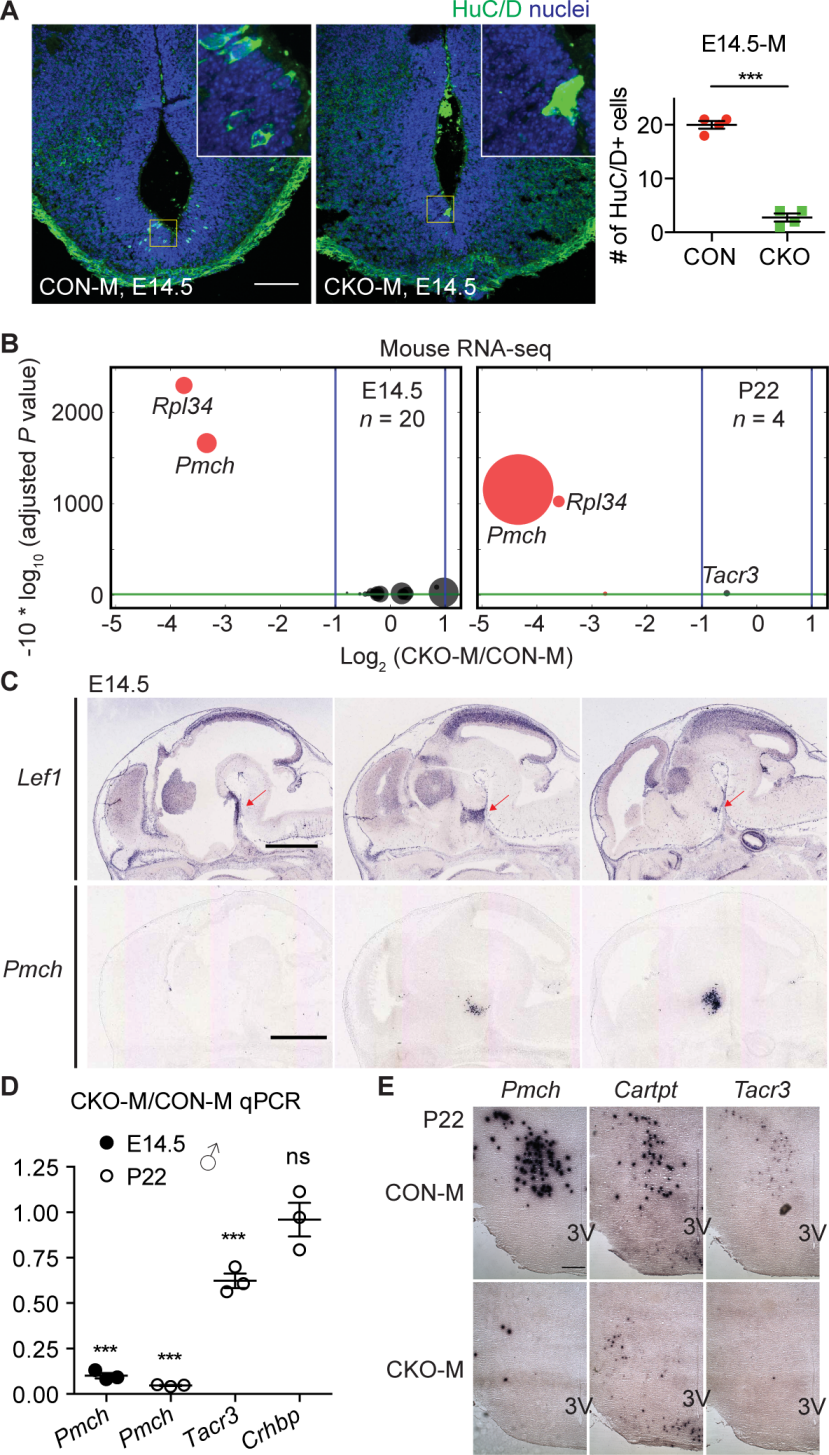
Hypothalamic Lef1 is required for *Pmch*+ neuron formation in mice. (A) Immunostaining of HuC/D+ cells in the hypothalamic ventricular zone of E14.5 CON-M and CKO-M, with quantification shown on the right (*n* = 4). Images are z-projections of 16 μm confocal optical slices, shown with dorsal side on top, and higher magnification views of yellow squares in the insets. (B) Volcano plot of mouse RNA sequencing (RNA-seq) shows differentially expressed genes in the hypothalamus of CKO-M compared to CON-M at E14.5 (left) and P22 (right), using the same format as in Fig 2A. (C) E14.5 sagittal in situ hybridization images (www.genepaint.org) show expression of *Lef1* (red arrows) and *Pmch* in the wild-type (wt) hypothalamus [26]. (D) Quantitative real-time PCR (qPCR) analysis for male shows hypothalamic gene expression in E14.5 and P22 CKO-M relative to CON-M. (E) P22 coronal in situ hybridization images show expression of *Pro-melanin concentrating hormone* (*Pmch*), *CART prepropeptide* (*Cartpt*), and *Tachykinin receptor 3* (*Tacr3*) in the lateral hypothalamus. 3V, third ventricle. Data are mean ± SEM. ***P < 0.001, ns. *P* > 0.05 by unpaired Student *t* tests. Scale bars: 400 μm in (C); 30 μm in (E). Raw data can be found in S1 Data.

Consistent with the neurogenesis defect we observed in zebrafish, we found fewer HuC/D+ cells in the mouse hypothalamic ventricular zone in *Lef1*^*CKO*^ embryos at E14.5 (Fig 5A). Importantly, this effect was restricted to coronal sections in which endogenous Lef1 is expressed (S5B Fig). To identify Lef1-dependent genes in the mouse hypothalamus, we performed RNA-seq analysis of hypothalami dissected from E14.5 *Lef1*^*CON*^ and *Lef1*^*CKO*^ embryos, and surprisingly identified only 1 protein-coding gene that mapped to a unique locus with an AdjP <0.1 and a fold change >2, *Pmch* (Fig 5B and S5 Table). *Pmch* expression normally overlaps with *Lef1* in the premammillary hypothalamus, and extends into the lateral hypothalamus (Fig 5C) [17,26]. We confirmed loss of *Pmch* expression in E14.5 *Lef1*^*CKO*^ embryos by quantitative real-time PCR (qPCR) and immunostaining (Fig 5D and S5D and S5E Fig). The only other significantly affected protein-coding gene identified by RNA-seq, *Ribosomal Protein L34* (*Rpl34*) (Fig 5B, S5 and S6 Tables), is a repetitive processed pseudogene that could not be conclusively mapped to a single genomic locus, although one copy is located adjacent to *Lef1*.

Reduced *Pmch* expression in *Lef1*^*CKO*^ embryos was unexpected because its orthologs were not significantly affected in RNA-seq analysis of zebrafish *lef1* mutants (S2 Table). To determine if any Lef1-dependent genes were conserved with zebrafish later in development, we performed another RNA-seq analysis at postnatal day (P) 22, when *Lef1*^*CKO*^ mice begin to exhibit a growth defect (Fig 4A). In this experiment, we identified only 2 affected protein-coding genes mapped to unique loci with an AdjP <0.1: *Pmch* and *Tachykinin receptor 3* (*Tacr3*) (Fig 5B, S6 Table). *Tacr3* is known to be co-expressed in *Pmch*+ neurons, along with *CART prepropeptide* (*Cartpt*) [27]. We confirmed their reduced expression in the lateral hypothalamus of P22 *Lef1*^*CKO*^ mice by qPCR and in situ hybridization (Fig 5D and 5E and S5E Fig), consistent with loss of *Pmch*+ neurons. Decreased body weight observed after ablating *Pmch*+ neurons [28,29] may therefore be related to an anxiolytic role for these cells [12], which is further supported by characterization of their inputs and activity [30].

Orthologs of multiple Lef1-dependent anxiety-related genes in zebrafish are expressed near *Lef1* in the mouse hypothalamus, such as *Pde9a* and *Nitric oxide synthase 1* (*Nos1*) at E14.5 [26], and *Crhbp* and *Histidine decarboxylase* (*Hdc*) in adults [16]. However, RNA-seq analysis indicated that expression of these genes was Lef1-independent in mice (S5 and S6 Tables), and we confirmed this result for *Crhbp* by qPCR and in situ hybridization (Fig 5D and S5E and S5F Fig). In addition, we confirmed that expression of zebrafish *pmch* orthologs [31] does not depend on Lef1 at either 3 dpf or 15 dpf (S6A–S6C Fig). While we cannot rule out the possibility that our RNA-seq analysis of the mouse hypothalamus lacked the sensitivity to identify other conserved Lef1-dependent genes, it is clear that the identity of Lef1-dependent neurons relevant for anxiety differs between zebrafish and mice.

### Lef1 dependence of *crhbp* expression is conserved between zebrafish and *Drosophila*

**Fig 6:**
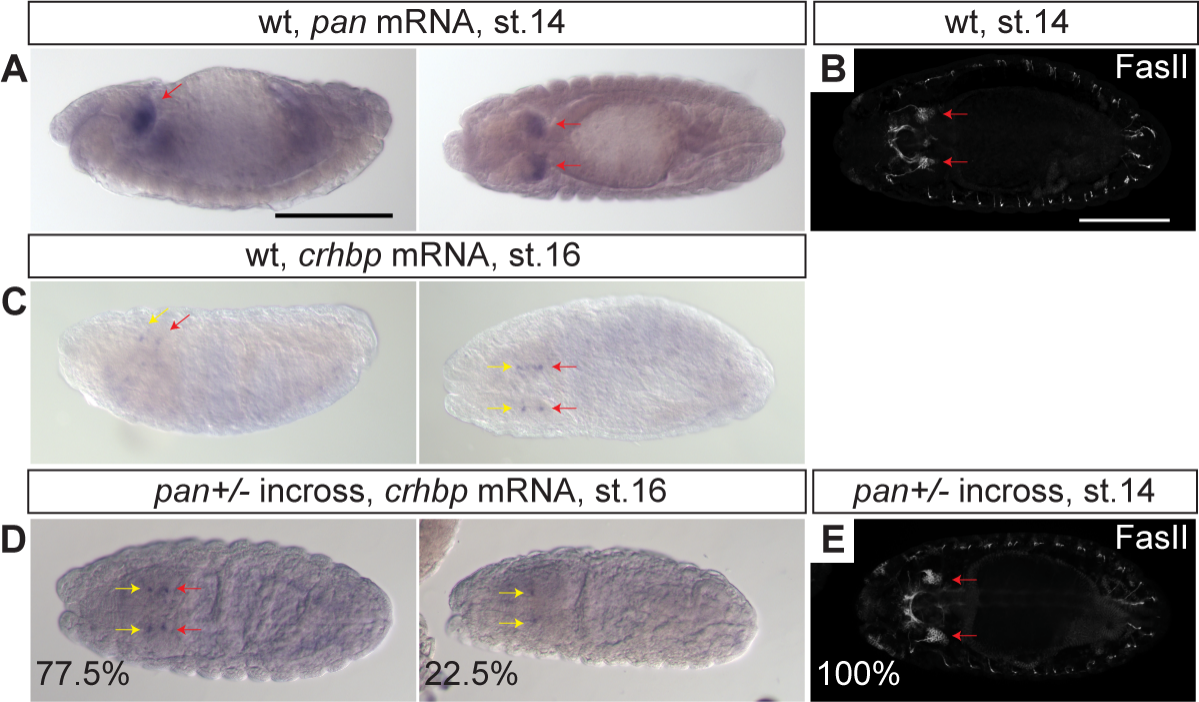
Loss of *Drosophila corticotropin-releasing hormone binding protein* (*crhbp*) expression in *pangolin* (*pan*) mutants. (A-E) Whole mount in situ hybridization for the *lef1* ortholog *pan* (A) and *crhbp* (C and D), and immunostaining for the pars lateralis (PL) marker FasII [32] (B and E) were performed in *Drosophila* wild-type (wt) embryos (A-C) and offspring from a *pan+/-* incross (D and E). Percentage of embryos with representative phenotype is displayed in (D) (*n* = 142) and (E) (*n* = 25). Confocal z-projections are shown in (B) and (E). All are representative images for at least 3 embryos. Left images in (A) and (C) are lateral views with dorsal side on top, and the other images are dorsal views. All images have anterior side on the left. Red and yellow arrows indicate the PL and pars intercerebralis (PI), respectively. Scale bars: 150 μm.

Interestingly, many Lef1-dependent genes in zebrafish encoding components of anxiety-mediating transmitter pathways, such as GABA, 5-HT, and CRH (Fig 2B), have a conserved function in *Drosophila* anxiety-like behavior [1]. Therefore, we hypothesized that hypothalamic Lef1-dependent neurons in zebrafish may represent an evolutionarily ancient pathway. The *Drosophila* pars intercerebralis (PI) and pars lateralis (PL) represent neuroendocrine organs equivalent to the vertebrate rostral hypothalamus and Hc, respectively [32]. In *Drosophila*, a single Lef/Tcf family member, *pangolin* (*pan*), functions as a Wnt activator [33,34]. Consistent with our hypothesis, we detected specific *pan* expression at stage 14 and the *crhbp* ortholog *CG15537* expression at stage 16 in the *Drosophila* PL primordium [32] (Fig 6A–6C). Furthermore, we observed a loss of *crhbp* expression in the PL of *pan* mutants [34] at stage 16, despite intact expression in the PI and normal PL morphology (Fig 6C–6E). *Drosophila crhbp* in the PL may also be anxiolytic by inhibiting CRH/CRH-like diuretic hormone in the PI [1,32,35], thus these results support a relationship between neuroendocrine Lef1 function and the development of anxiolytic Crhbp+ neurons dating back to a common bilaterian ancestor. By contrast, *Pmch* is a vertebrate specific gene, and Lef1-dependent *Pmch+* neuronal circuitry in mice may reflect a more recent mammalian divergence that co-evolved with new brain structures [36].

### Coordinated expression of *PMCH* and *CRHBP* in the human hypothalamus

**Fig 7:**
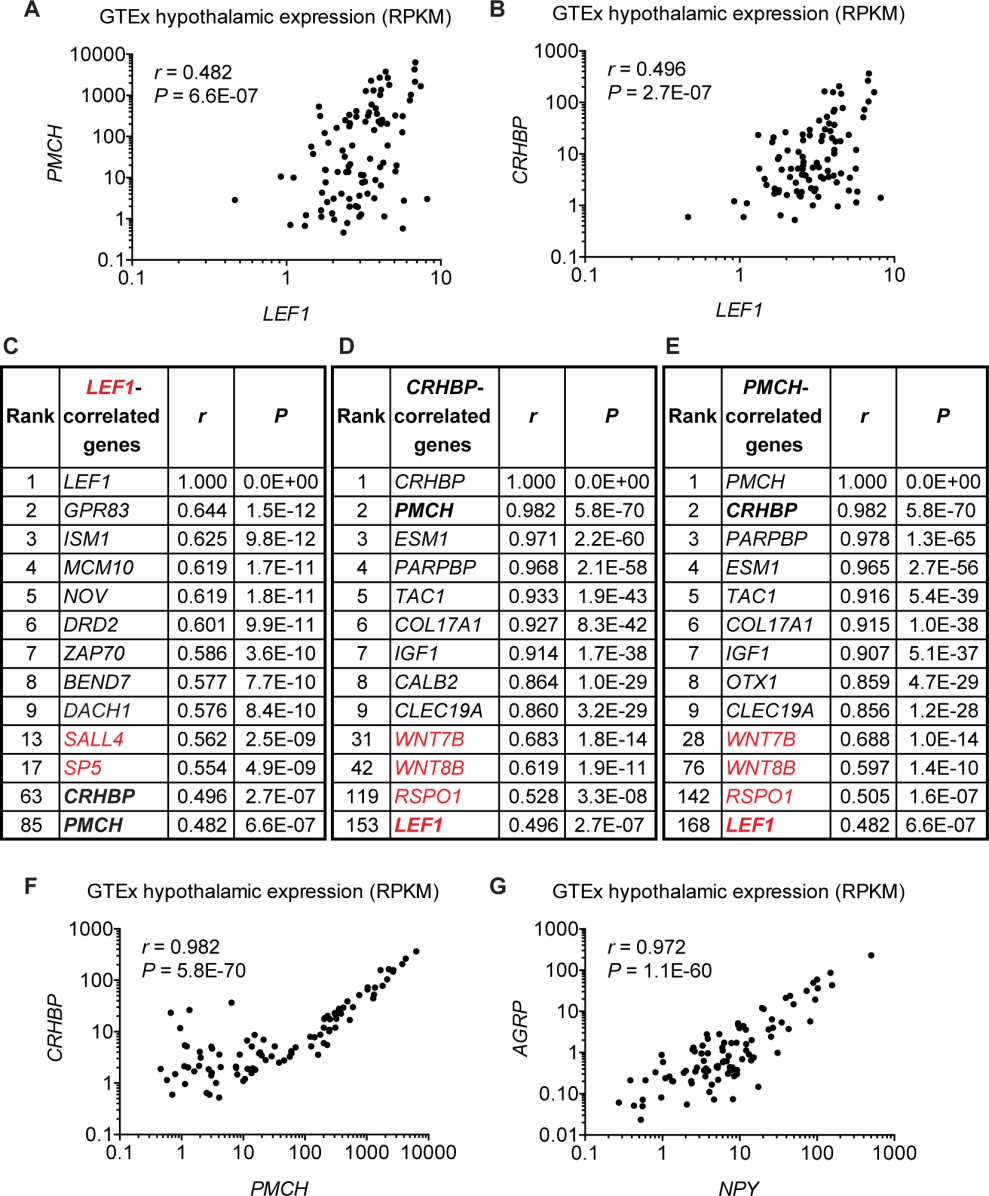
Correlation analysis in the human hypothalamus. (A-G) Pearson correlations for hypothalamic gene expression among 96 postmortem human samples obtained from the Genotype-Tissue Expression (GTEx) project [39]. All the Pearson’s *r* and *P* values were calculated between 2 genes, and displayed in the graphs or tables after sorting by *r* values. Correlation expression profiles are shown for gene pairs *Pro-melanin concentrating hormone* (*PMCH*) versus *LEF1* (A), *Corticotropin-releasing hormone binding protein* (*CRHBP*) versus *LEF1* (B), *CRHBP* versus *PMCH* (F), and *Agouti-related protein* (*AGRP*) versus *Neuropeptide Y* (*NPY*) (G), with reads per kilobase of transcript per million mapped reads (RPKM) at log_10_ scale used on both axes. Note that 1 data point (*NPY*: 0.1395; *AGRP*: 0) was not included in (G) due to the inability of plotting a 0 value on the logarithmic axis. Three tables of correlated genes for *LEF1* (C), *CRHBP* (D), and *PMCH* (E) list the top 9 positively correlated genes plus selected genes, including those involved in canonical Wnt signaling labeled in red. See the full list in S8 Table.

Our animal models suggest that in humans Lef1 may also regulate the formation of Pmch+ and/or Crhbp+ hypothalamic neurons. To test this hypothesis, we compared the hypothalamic RNA-seq transcriptomes of 96 human individuals from the GTEx project [37] (S7 Table). Despite the fact that these data did not include prenatal samples, we found that expression of *PMCH* and *CRHBP* are both moderately correlated with *LEF1*, which is expressed at a relatively low level in the adult human hypothalamus (Fig 7A and 7B). Notably, *PMCH* and *CRHBP* were both within the top 100 *LEF1*-correlated genes, along with known Wnt targets such as *Sal-like protein 4* (*SALL4*) [38] and *SP5* [11] (Fig 7C and S8 Table).

In the course of this analysis, we noticed similar correlation profiles for *CRHBP* and *PMCH* (Fig 7A and 7B), suggesting a possible expression correlation between these 2 genes. Surprisingly, we found *CRHBP* and *PMCH* to be the most highly correlated genes with each other (Fig 7D–7F and S8 Table), a relationship that has never been reported previously. Among the top 200 *PMCH*- or *CRHBP*-correlated genes, we also found 2 Wnt ligands and 1 Wnt co-activator: *R-Spondin 1* (*RSPO1*) [40] (Fig 7D and 7E). As a comparison, *AGRP* is the most highly correlated gene with *Neuropeptide Y* (*NPY*) (Fig 7G and S8 Table), consistent with their co-expression in the same hypothalamic neurons [41]. Interestingly, while *Pmch* and *Crhbp* are expressed in different regions of the mouse hypothalamus [16], they are expressed in the same hypothalamic nuclei in another primate, the marmoset according to the Marmoset Gene Atlas (https://gene-atlas.bminds.brain.riken.jp). Importantly, the results of all our correlation analyses are recapitulated on GeneNetwork (www.genenetwork.org) [42], which imported an older version of GTEx’s datasets and calculated Pearson correlation across a population (See Materials and methods). Together these data suggest co-expression of *PMCH* and *CRHBP* in the primate hypothalamus and potential regulation by *LEF1*-mediated Wnt signaling in humans.

## Discussion

**Fig 8:**
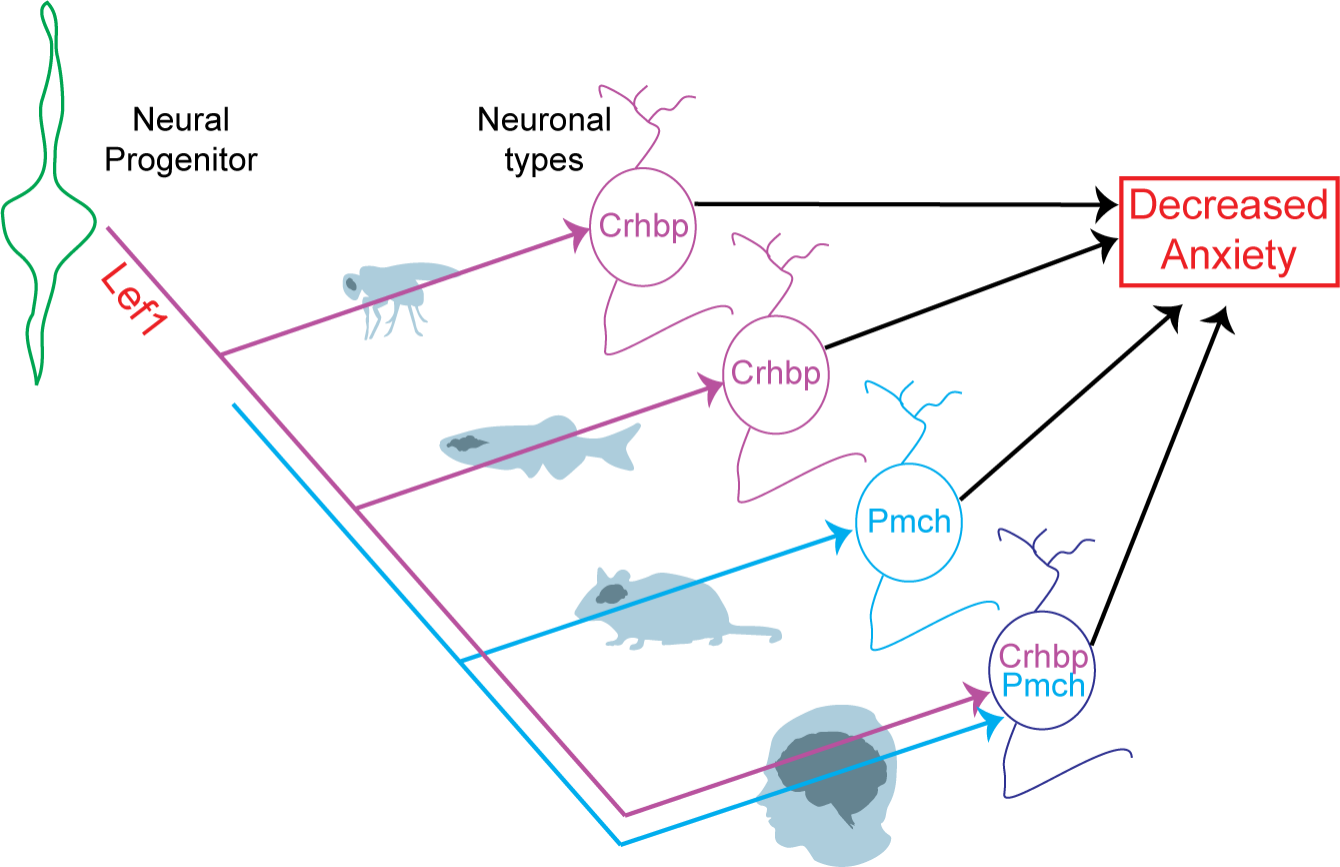
Mechanism of Lef1-mediated Wnt signaling in hypothalamic neurogenesis and anxiety. Lef1-mediated Wnt signaling plays an evolutionarily conserved role in hypothalamic neurogenesis that inhibits anxiety. However, the underlying molecular and cellular mechanisms can vary between organisms.

In this study, we demonstrate that Lef1-mediated hypothalamic Wnt signaling plays an evolutionarily conserved role in regulating the formation of anxiolytic neurons (See Fig 8 for summary). In zebrafish *lef1* mutants, neural progenitors fail to differentiate and undergo apoptosis, resulting in a smaller Hc (alternatively named the hypothalamic posterior recess, the posterior part of the paraventricular organ, or the caudal zone of the periventricular hypothalamus [4,43,44]). Any or all of the 20 anxiety-related genes that are misregulated in the zebrafish mutant (Fig 2B) may contribute to the behavioral phenotypes that we observe. Likewise, our data do not conclusively prove that *crhbp*+ neurons, or indeed any individual Lef1-dependent neuronal populations, mediate the effect of Lef1 on anxiety. Such a conclusion would require either rescue of the *lef1* mutant phenotype by restoration of missing neurons, or phenocopy by specific ablation of the cells. However, the specific loss of *Pmch*+ neurons in our mouse conditional knockout (Fig 5B), combined with the unexpected expression correlation between *PMCH* and *CRHBP* in the human hypothalamus (Fig 7F), is consistent with a common role for these 2 genes in behavior. While we also cannot rule out the possibility that *Lef1* mutants may have other behavioral defects, genes that are known to regulate other hypothalamus-driven behaviors, such as *Npy*, *Agrp*, *Pomc*, and *Hcrt*, are unaffected in our mutants (S2, S5 and S6 Tables). In addition, pure assessment of other behaviors cannot distinguish a direct phenotype from an anxiety-related secondary phenotype.

While the major product of Pmch, melanin-concentrating hormone (MCH), is an anxiolytic factor in teleosts [45], studies in mammals have reported it to be either anxiolytic, anxiogenic or having no effect [46,47]. In addition, the Pmch propeptide makes at least 2 more neuropeptides, neuropeptide-glutamic acid-isoleucine (NEI), and neuropeptide-glycine-glutamic acid (NGE), which are also involved in stress response and anxiety [48]. Germline *Pmch* mouse knockouts gain weight more slowly than controls, a phenotype originally attributed to decreased food intake [49]. However, on a different background strain, the same group reported that the knockout mice were not hypophagic, while retaining a growth phenotype [50]. Interestingly, all rodent models ablating *Pmch* [49–53] or *Pmch*+ neurons [28,29] exhibit a reduced growth rate. One possible underlying mechanism could be enhanced anxiety [12], which was not directly tested in any of these studies. Therefore, we hypothesize that in *Lef1*^*CKO*^ mice, loss of hypothalamic *Pmch*+ neurons is responsible for elevated anxiety, leading to a secondary growth phenotype.

Our data suggest that the gene expression and neuronal subtypes dependent on Lef1 can change during evolution while maintaining a common behavioral output. While transcriptional networks can undergo rapid rewiring at the level of enhancer binding sites during yeast, insect and mammalian evolution [54,55], the direct transcriptional targets of Lef1 mediating hypothalamic neurogenesis are still unknown. We have identified Tcf/Lef consensus binding sites in zebrafish and mouse *Crhbp* and *Pmch* loci, but it remains important to determine whether these 2 genes are direct targets of Lef1, or are instead lost as a secondary result of neurogenesis defects in mutants. In either case, it will also be useful to understand the circuitry of Lef1-dependent neurons. While the targets of *Crhbp*+ neurons in *Drosophila* and zebrafish are unknown, the projections of *Pmch*+ neurons in the hypothalamus of mice and other mammals are well characterized, and the regulation of these circuits by Lef1 in these species may be linked to anatomical and functional expansion of target brain regions such as the cortex [36]. Importantly, the coordinated expression of *CRHBP* and *PMCH* in the human hypothalamus suggests that they may be co-expressed in a single neuronal cell type.

Loss of other genes important for hypothalamic neurogenesis has been shown to affect behavior [2]. Interestingly, mice lacking hypothalamic *Dbx1* also exhibit a loss of *Pmch*+ neurons along with other populations [56]. In that study, *Lef1*-expressing hypothalamic nuclei were hypothesized to regulate innate behaviors outside the hypothalamic-pituitary-adrenal (HPA) axis, partly due to the observation of expanded Wnt activity in *Dbx1* knockout animals. However, because our work demonstrates that *Lef1* is in fact required for the genesis of *Pmch*+ neurons and for HPA-related behaviors, an alternative explanation is that *Dbx1* functions in a parallel pathway to *Lef1*.

Together these results identify Wnt signaling as a link between brain development and function that allows essential behaviors to be maintained even as anatomical structures change through evolution. In addition, given the function for hypothalamic Wnt signaling in regulating postembryonic zebrafish neurogenesis [4], and the continuous expression of Lef1 in the hypothalamus of fish (S2D Fig) and mammals [16] throughout life, it would be interesting to test a possible contribution to adult behavior using temporal conditional knockout models. While Wnt signaling in the mammalian hippocampus and nucleus accumbens has been associated previously with anxiety and depression [57,58], our data demonstrate a novel requirement for pathway activity in a brain region that is highly conserved throughout the vertebrate lineage, and may prove useful for the diagnosis and treatment of hypothalamus-related anxiety disorders.

## Materials and methods

### Ethics statement

All experimental protocols were approved by the University of Utah Institutional Animal Care and Use Committee and were in accordance with the guidelines from the National Institutes of Health. Approval number: 16–09011. Zebrafish were euthanized by ice water immersion. Mice were euthanized by CO2 or ketamine/xylazine.

### Subjects: Zebrafish

Zebrafish (*Danio rerio*) were bred and maintained in a 14:10 hour light/dark cycle as previously described [59]. Zebrafish per tank were fed with similar amount of food and treated by the staff who were blinded to the experiments. Wt strains were *AB. The following mutant and transgenic strains were used: *lef1*^*zd11*^ [4], *Tg(top*:*GFP)*^*w25*^ [7], *Tg(dlx6a-1*.*4dlx5a-dlx6a*:*GFP)*^*ot1*^ [60], *Tg(h2afv*:*GFP)*^*kca6*^ [61], *Tg(th2*:*GFP-Aequorin)*^*zd201*^ [8], *p53*^*e7*^ [62]. *lef1*-/- homozygous mutants were identified between 3 dpf and 10 dpf by DASPEI staining as described previously [15] and at or after 15 dpf by loss of caudal fin [4]; wt and heterozygous siblings were used as controls. All the zebrafish were from at least 1 single-pair breeding. Genotyping was done as described before for *lef1*^*zd11*^ [4] and *p53*^*e7*^ [63], except primers used for *lef1*^*zd11*^ (forward primer: 5ʹ-CACTCTCTCCAGCCCAACATT-3ʹ, reverse primer: 5ʹ-TGTTACTGTTGGGACTGATTTCTG-3ʹ).

### Subjects: Mice

Male and female C57BL/6J mice (*Mus musculus*) were group-housed with 2–5 mice per cage in a reverse 12 hour light/dark cycle with ad libitum access to food and water. Mice were 19–20 and 15–20 weeks old at the time of behavioral tests for male and female animals, respectively. *Ai9* reporter *Rosa*^*tdTomato*^ (line 007905) [22], *Nkx2-1*^*Cre*^ (line 008661) [21], and *TCF/Lef*:*H2B-GFP* mice (line 013752) [23] were purchased from Jackson Laboratories. *Lef1*^*flox/flox*^ mice were provided by HHX [20]. All strains were maintained on a C57BL/6J background except *TCF/Lef*:*H2B-GFP* mice, which were originally on a C57BL/6 × 129 background. Male *Nkx2-1*^*Cre/Cre*^*;Lef1*^*flox/+*^ and female *Lef1*^*flox/flox*^*;Rosa*^*tdTomato/tdTomato*^ mice were used to generate conditional knockout (*Lef1*^*CKO*^: *Nkx2-1*^*Cre/+*^*;Lef1*^*flox/flox*^*;Rosa*^*tdTomato/+*^) and control (*Lef1*^*CON*^: *Nkx2-1*^*Cre/+*^*;Lef1*^*flox/+*^*; Rosa*^*tdTomato/+*^) offspring. Females breeders were maintained by inbreeding. Male breeders were maintained by interbreeding *Nkx2-1*^*Cre/Cre*^*;Lef1*^*+/+*^ and *Nkx2-1*^*Cre/Cre*^*;Lef1*^*flox/+*^ for no more than 5 generations to avoid potential artifacts caused by Cre homozygous inbreeding [64]. In occasional litters, *Ai9* reporter expression was observed throughout the body of approximately 10% of experimental animals, consistent with published literature [21]; such animals were not used for experiments. All the mice were from at least 3 litters unless otherwise noted. Sex at E14.5 was determined by genotyping by Jarid 1c [65]. When generating experimental mice for body weight measurement and behavioral tests, each litter was culled to 8 pups at P0. Genotyping for *Rosa*^*tdTomato*^ and *TCF/Lef*:*H2B-GFP* animals was done according to available Jackson Laboratory protocols for these strains. Genotyping for *Nkx2-1*^*Cre*^ mice was done using primers for Cre recombinase detection (forward primer: 5ʹ-ATGCTTCTGTCCGTTTGCCG-3ʹ, reverse primer: 5ʹ-CCTGTTTTGCACGTTCACCG-3ʹ). Genotyping for *Lef1*^*flox*^ mice was done using primers contributed by HHX (forward primer: 5ʹ-GCAGATATAGACACTAGCACC-3ʹ, reverse primer: 5ʹ-TCCACACAACTAACGGCTAC-3ʹ).

### Subjects: *Drosophila*

Canton-S wild-type and *pan*^*2*^ mutant (BL4759) *Drosophila melanogaster* strains were obtained from Bloomington Stock Center.

### Zebrafish transplantation experiments

At the sphere stage, 10–50 blastula cells from donor embryos were transplanted using a glass micropipette into the dorsal side of shield stage host embryos, 20–40 degrees from the animal pole, representing the hypothalamus anlage [66]. Embryos were then raised to 5 dpf for immunohistochemistry. Donor and host embryos were retained for genotyping to identify *lef1* mutants.

### BrdU labeling

Four dpf zebrafish embryos were incubated in E3 media containing 10 mM BrdU (Sigma-Aldrich, St. Louis, MO) at 28.5°C for indicated time before being washed in E3 media for at least 3 times.

### Immunohistochemistry: Zebrafish

Embryos and larvae were fixed in 4% paraformaldehyde (PFA) for 3 hours at room temperature (RT) or overnight (O/N) at 4°C followed by brain dissection. Brains were either dehydrated in methanol and stored at −20°C, or immediately processed for immunohistochemistry. For 3 dpf embryos, 5% sucrose was included in the fixative to ease dissection. Brains were treated with 0.5 U dispase (Gibco #17105–041) in 2% PBST (PBS/2% Triton X-100) for 60 minutes at RT. For BrdU, PCNA, pH3 or Caspase-3 staining, brains were washed in water for 5 minutes twice, followed by incubation in 2 N HCl for 60 minutes at RT, followed by 2 more water washes. Brains were then blocked in 5% to 10% goat serum in 0.5% PBST for 60 minutes at RT. Embryos were incubated in primary antibodies in block O/N at 4°C and secondary antibodies and Hoechst 33342 (Life Technologies, H3570) in block O/N at 4°C before mounting in Fluoromount-G (SouthernBiotech, Birmingham, AL) with the ventral hypothalamus facing the coverslip. Primary antibodies were all used at 1:500 dilution except as noted: chicken anti-GFP (Aves Labs, GFP-1020), rabbit anti-GFP (Molecular Probes, A11122), mouse anti-HuC/D (Molecular Probes, A21271), rabbit anti-5-HT (ImmunoStar, 541016), rabbit anti-pH3 (1:400, Cell Signaling, 9713), rabbit anti-active Caspase-3 (BD Pharmingen, 559565), rabbit anti-BLBP (Abcam, ab32432), mouse anti-PCNA (Sigma, P8825), and chicken anti-BrdU (ICL, CBDU-65A-Z). Secondary antibodies were all used at 1:500 dilution: goat anti-mouse Alexa Fluor 448 (Invitrogen, A11001), goat anti-rabbit Alexa Fluor 488 (Invitrogen, A11008), donkey anti-chicken Alexa Fluor 488 (Jackson ImmunoResearch, 703-545-155), goat anti-rabbit cy3 (Jackson ImmunoResearch, 111-165-003), goat anti-mouse cy3 (Jackson ImmunoResearch, 115-165-003), goat anti-mouse Alexa Fluor 647 (Invitrogen, A21235), goat anti-rabbit Alexa Fluor 647 (Invitrogen, A21244), and goat anti-chicken Alexa Fluor 647 (Invitrogen, A21449). Hoechst 33342 (1:10,000) was used to stain nuclei. All the primary antibodies were validated previously [4,67].

### Immunohistochemistry: Mice

E14.5 embryo heads were dissected in PBS and fixed in 4% PFA at RT for 1.5 hours or O/N at 4°C. Brains were dissected and cryoprotected in 15% and then 30% sucrose, embedded in OCT, and stored at −80°C. Brains were cryosectioned at a thickness of 16 μm, air dried and stored at −80°C. Air-dried sections were then washed in PTW (PBS+0.1% Tween 20) 3 times, followed by permeabilization in 0.25% PBST for 5 minutes and blocking in 10% goat serum in PTW for 60 minutes. Sections were incubated in primary antibodies in blocking solution O/N at 4°C and secondary antibodies in blocking solution for 2 hours at RT, followed by Hoechst 33342 stain for 10 minutes at RT before mounting in Fluoromount-G. Antibodies used were as described above except rabbit anti-LEF1 (1:200, Cell Signaling, 2230), goat anti-PMCH (1:500, Santa Cruz, sc14509) and donkey anti-goat Alexa Fluor 647 (1:400, Invitrogen, A21447). All primary antibodies were validated by absence of staining in *Lef1*^*CKO*^ animals. For HuC/D staining, incubation for 30 minutes in 0.5 U dispase was performed in 0.25% PBST.

### Immunohistochemistry: *Drosophila*

*Drosophila* immunohistochemistry was performed as previously described [68] except that a fluorescent secondary antibody was used. Antibodies used were as described above except mouse anti-FasII (1:5, DSHB, 1D4), which was validated previously [32].

### Probes for in situ hybridization

In situ hybridization probes were made by a clone-free method as described previously [69,70], with DNA templates purified using Zymo Research DNA Clean & Concentrator-5 kit. Primers were designed by Primer-BLAST [71] except for mouse genes with primer sequences available from the Allen Brain Atlas (ABA) [16] or GenePaint Atlas [26]. A full list of primers used to make probes is in S9 Table. cDNA made from 3 dpf zebrafish embryos, P2, and P60 mouse hypothalamus, and adult *Drosophila* (gift from C. Thummel) was used as the initial template for PCR to generate T7 promoter-containing DNA. RNA probes for zebrafish *lef1* [72] and *axin2* [73] were previously described. The RNA probe for *Drosophila pan* was generated from the *Drosophila* Gene Collection T7 promoter-containing cDNA GM04312 [74].

### Whole mount in situ hybridization: Zebrafish

Zebrafish whole mount in situ hybridization was performed as described previously [75] except that 15 dpf and adult zebrafish were fixed in 4% PFA O/N at 4°C followed by washing in PBS and brain dissection. All tissues were treated for 30 minutes with 10 μg/ml Proteinase K. Pigmented embryos were bleached in 1% H_2_O_2_/5% formamide/0.5× SSC O/N at RT after in situ hybridization. 3 dpf embryos and postembryonic brains were imaged in 100% glycerol and PBS, respectively. For automated whole mount in situ hybridization, all steps following probe hybridization and before color reaction were performed using a BioLane HTI (Intavis, Chicago, IL).

### Section in situ hybridization: Mice

Twenty-five μm brain cryosections were collected and post-fixed as previously described [76] (http://developingmouse.brain-map.org/docs/Overview.pdf). In situ hybridization was then performed as described [77].

### Whole mount in situ hybridization: *Drosophila*

*Drosophila* whole mount in situ hybridization was performed as described previously [68].

### Body length: Zebrafish

Zebrafish from a single home tank were anesthetized using tricaine (Sigma-Aldrich, St. Louis, MO) in shallow water. Images were acquired of immobilized, non-overlapping fish with a ruler for scale. Body length was calculated by measuring the distance between the mouth and the anterior edge of the tail fin, using ImageJ.

### Novel tank diving test

Five fish from *lef1+/-* incrosses were raised per tank starting at 5 dpf. *lef1* mutants and controls were separated at 15 dpf. Novel tank diving tests [13] were performed on 16 dpf larvae during the early afternoon of the same days, before *lef1* mutants start to display surfacing behavior at 20 dpf. Novel rectangular tanks (16.6 cm × 9.5 cm × 12.3 cm) were illuminated by a centered white light, and videos were acquired with a mounted Nokia Lumia 640 phone 1080p camera. For each experiment, single mutant and control larvae were netted and then removed simultaneously from their home cages and transferred to novel tanks with identical water volume. The order of netting mutant and control fish was rotated between trials. Videos were viewed in MPlayerX to manually analyze the latency of larvae to enter the upper half of the tank after initial sinking. Videos were then imported and analyzed using Ethovision XT version 11.5 (Noldus, Leesburg, VA) during the initial exploration phase, with a tracking period of 2 minutes beginning 1 minute after release into the novel tank to decrease water agitation resulting from netting. Videos were also analyzed after the initial exploration phase with a tracking period during the 4 to 6 minute interval. Tracks were analyzed for distance travelled, time in upper half of the tank and time of immobility.

### Body weight: Mice

All pups were weaned at P21 immediately following the first weighing. Pups weighing less than 6.5 g were excluded from analysis. All mice were weighed during the morning of the same days of the following weeks.

### Behavior tests: Mice

Group-housed mice were allowed to acclimate to the animal facility for behavioral tests 9 days after an on-campus transfer. Each mouse was handled daily for 2 minutes, during midmorning for 7 days before commencement of behavioral testing using the cupped hand method [78]. To avoid behavioral variation caused by female estrous cycle [79], a vaginal lavage procedure was done after daily handling for estrous phase evaluation for 7 days, as previously reported [80]. Female mice in their proestrus or estrus phases were collectively grouped as “Estrus” and females in their metestrus and diestrus phases were collectively grouped as “Diestrus.” All mice were acclimated to the behavior room for 1 hour under red light (69 lux) before commencement of tests. Open field and EPM behavioral tests were performed in order, once daily for 2 days, from 9 am to 5 pm. The experimenter was blinded to genotype.

### OFT

Each mouse was placed in a circular plexiglass chamber (4.5” diameter × 3” height) located inside an illuminated (330 lux) circular open field arena (110 cm diameter) and allowed to acclimate for 1 minute to decrease movement bias resulting from experimenter handling. After 1 minute, the plexiglass chamber was removed from the arena, and the mouse was allowed to freely explore the arena for 10 minutes. Movement was video recorded and analyzed using Ethovision version 9 (Noldus, Leesburg, VA).

### EPM

The EPM apparatus was elevated 60 cm from the floor, having 2 open arms (35 cm × 5 cm) and 2 closed arms (35 cm × 16 cm) connected by a central platform (5 cm × 5 cm). The EPM was illuminated by a white light (205 lux) at the center platform. Each mouse was placed in a rectangular opaque white plexiglass chamber (2” × 3” × 5”) located on the center platform, and allowed to acclimate for 1 minute before commencement of the test. The white chamber was removed and the mouse was allowed to freely explore the EPM for 5 minutes. Behavior was video recorded and analyzed using Ethovision version 9 (Noldus, Leesburg, VA).

### RNA-seq: Zebrafish

Embryos were fixed for 1.5 hours in 4% PFA/5% sucrose in PBS at RT, followed by whole hypothalamus dissection with super-fine forceps (FST, 11252–00). For each biological replicate, 28 to 38 dissected hypothalami were pooled for *lef1* mutant and control samples from at least 1 single-pair breeding. RNA was extracted using a RecoverAll Total Nucleic Acid Isolation Kit for FFPE (Ambion, AM1975) according to the manufacturer’s instructions. Three biological replicates were obtained on different days from offspring of different breedings. A total of 300 ng RNA per sample was submitted to the High Throughput Genomic Core at the University of Utah for RNA quality control by High Sensitivity R6K ScreenTape, RNA concentration by vacuum drying, cDNA library prep by Illumina TruSeq Stranded RNA Kit with Ribo-Zero Gold and sequencing by HiSeq 50 Cycle Single-Read Sequencing version 3. RNA-seq was analyzed by the Bioinformatics Core at the University of Utah. A transcriptome reference was created by combining GRCz10 chromosome sequences with Ensembl build 84 splice junction sequences generated with USeq (version 8.8.8) MakeTranscriptome. RNA-seq reads were mapped to the GRCz10 zebrafish transcriptome reference using Novoalign (version 2.08.03). Splice junction alignments were converted back to genomic space using USeq SamTranscriptomeParser. USeq DefinedRegionDifferentialSeq was used to generate per gene read counts, which were used in DESeq2 to determine differential expression. RNA-seq graph in Fig 2A was made by IPython Notebook with package NetworkX.

### RNA-seq: Mice

E14.5 and P22 nonweaned male *Lef1*^*CON*^ and *Lef1*^*CKO*^ hypothalami were dissected using a fluorescent microscope in ice-cold PBS, while tail tissue was retained for genotyping. E14.5 tissues were immediately immersed in RNAlater (Thermo Fisher, Waltham, MA) and stored at 4°C for up to 7 days until RNA extraction. P22 tissues dissected from at least 2 litters were immediately homogenized in TRIzol (Thermo Fisher, Waltham, MA) and stored at −80°C. Three biological replicates were prepared from either 5 pooled hypothalami (E14.5) or a single hypothalamus (P22) from *Lef1*^*CON*^ and *Lef1*^*CKO*^ mice, and RNA was extracted on the same day using TRIzol followed by purification with an RNeasy Mini Kit (Qiagen, Hilden, Germany) and on-column DNase digestion (Sigma-Aldrich, St. Louis, MO). One μg of RNA per sample was submitted to the High Throughput Genomic Core at the University of Utah for RNA quality control with Agilent RNA ScreenTape, cDNA library prep with Illumina TruSeq Stranded RNA Kit with Ribo-Zero Gold, and sequencing using HiSeq 50 Cycle Single-Read Sequencing version 4. RNA-seq reads were mapped to GRCm38. Differential gene expression analysis and graph plotting were carried out using the same methods as for zebrafish RNA-seq.

### qPCR

Three biological replicates of RNA from male and female mice were prepared as described above for RNA-seq. Two and a half μg RNA was used for cDNA synthesis with a SuperScript III Reverse Transcriptase kit (Invitrogen, Carlsbad, CA). qPCR was performed in triplicate using Platinum SYBR Green master mix (Invitrogen, Carlsbad, CA) on 96-well CFX Connect (Bio-Rad, Hercules, CA) plates or 384-well QuantStudio 12K Flex (Life Technologies, Durham, NC) plates at the Genomics Core at the University of Utah, according to manufacturer’s instructions. *Gapdh* was used to normalize quantification, and reverse transcriptase was omitted for controls. qPCR analysis was performed with the ΔΔC_t_ method to determine relative expression change [81]. Dissociation curve analysis was performed to confirm the specificity of amplicons. qPCR primers were designed from PrimerBank [82] as follows (forward primer first, reverse primer second, in 5ʹ to 3ʹ orientation with PrimerBank ID in the parentheses), *Pmch* (12861395a1): GTCTGGCTGTAAAACCTTACCTC, CCTGAGCATGTCAAAATCTCTCC; *Tacr3* (10946720a1): CTGGGCTTGCCAGTGACAT, CGCTTGTGGGCCAAGATGAT; *Crhbp* (162287189c2): CTTACCCTCGGACACTTGCAT, GGTCTGCTAAGGGCATCATCT.

### Image analysis and cell counting

Fluorescent images of dissected zebrafish and mouse brains were obtained with an Olympus FV1000 confocal microscope at the Cell Imaging Core at the University of Utah. Z-stack images were all maximum intensity z-projections of 3 μm slices; single- or double-labeled cells were manually counted in FV1000 ASW 4.2 Viewer. All the zebrafish and mouse in situ hybridization images were obtained with an Olympus SZX16 dissecting microscope except those in Fig 5E, S2C Fig and S6B Fig, which were obtained with an Olympus BX51WI compound microscope. Two months post-fertilization (mpf) zebrafish images (S3A and S3B Fig) were acquired using a Leica MZ16 microscope. *Drosophila* in situ hybridization images were obtained with a Zeiss Axioskop.

### IPA

IPA (QIAGEN, Redwood City, CA) was performed with 129 mouse orthologs of the 138 zebrafish protein-coding genes identified from RNA-seq with AdjP <0.1 (S4 Table). Analysis was performed by the Bioinformatics Core at the University of Utah according to QIAGEN's instructions and “diseases and functions” were extracted from the software (S3 Table).

### Human correlation analysis

Publically available GTEx raw datasets were downloaded from www.gtexportal.org in April 2017 as a single file: GTEx_Analysis_v6p_RNA-seq_RNA-SeQCv1.1.8_gene_rpkm.gct.gz. Ninety-six hypothalamic samples were identified according to their specific strong *PMCH* expression, and extracted into S7 Table by IPython Notebook with packages gzip and xlwt. Pearson correlation was calculated by gene reads per kilobase of transcript per million mapped reads (RPKM) using IPython Notebook with function scipy.stats.stats.pearsonr, followed by result writing into S8 Table by IPython Notebook with package xlwt. The same Pearson correlation *r* values were confirmed using Excel’s CORREL function. A similar correlation result was obtained when searching for the top 200 correlated genes by Pearson on GeneNetwork (www.genenetwork.org) in April 2017. Several differences are noted between our analyses and GeneNetwork’s analyses. First, GeneNetwork imported an older version of GTEx’s datasets (GTEXv5 Human Brain Hypothalamus RefSeq [Sep15] RPKM log2). Second, GeneNetwork calculated Pearson correlation using RPKM log_2_ rather than RPKM in our case. Third, GeneNetwork calculated Pearson’s sample correlation across a population, with an adjustment across the genome, and also based on the number of the top correlated genes requested by the users; in our case, we calculated Pearson correlation between 2 genes, and simply ranked all the genes by their Pearson’s *r* values calculated for the gene of interest. Lastly, GeneNetwork’s imported older GTEx datasets had 102 hypothalamic samples, 6 among which were left out in current GTEx’s server. The complete overlapping of the 96 samples further confirmed our successful extraction of hypothalamic datasets from the GTEx project.

### Statistical analysis

No statistical methods were used to predetermine sample size. For behavioral assays, sample size was determined based on accepted practice. The experiments were not randomized. Due to visible phenotypes, the investigators were not blinded to outcome assessment except for whole mount in situ hybridization of zebrafish *lef1+/-* incrosses, *Drosophila pan+/-* incrosses, and mouse body weight and behavioral assays. Two-tailed unpaired Student *t* tests were performed for all statistical analysis, except mouse body weight (2-way ANOVA with repeated measures), using GraphPad Prism software version 6. Outliers were identified by Grubbs’ test for behavioral assays with significance assigned at *P* < 0.05 (alpha = 0.01). All the criteria for excluding data points were established prior to data collection.

## Supporting information

S1 Fig Lef1 promotes neurogenesis in the zebrafish caudal hypothalamus (Hc). (**A** and **B**) Hc size in control and *lef1* mutants (**A**) estimated by the area of confocal ventricular slice (**B**). Hc was defined as an oval indicated by red outline in (**B**). The lengths of a1, a2, b1, b2 in the representative image (**B**) were measured by ImageJ, and the area of the oval was calculated by the following equations: Estimated area = π*a*b/4; a = a1+a2; b = (b1+b2)/2. (**C**) Co-immunostaining of HuC/D and GABAergic lineage marker *dlx5/6*:*GFP* [83] in the 3 dpf Hc. Three confocal channel-split magnified images of the region depicted by the yellow rectangle are shown on the right. A representative image is shown for at least 3 embryos tested. (**D** and **E**) Immunostaining of *th2*:*GFP*+ (**D**) and BLBP+ cells (**E**) in the Hc of 3 dpf control and *lef1* mutant. Representative images are shown on the left, and quantifications are shown on the right. Higher magnification views of yellow rectangles in single channel are shown in the insets in (**E**). (**F**-**H**) Measurement of proliferation in the Hc of 5 dpf control and *lef1* mutant as shown by pH3+ (**F**) and PCNA+ cells (**G**; representative image on the left and quantification on the right; cells adjacent to the horizontal ventricle were counted), and 1 day BrdU labeling (**H**; schematic on the left). (**I**), BrdU pulse-chase (schematic on the left) to measure birth of 5-HT+ and ventricular HuC/D+ cells after 4 dpf. Data are mean ± SEM, except mean ± SD in (**A**) and (**I**). ***P < 0.001, **P < 0.01, *P < 0.05, ns. *P* > 0.05 by unpaired Student *t* tests. All images are confocal ventricular slices. All scale bars are 25 μm except 12.5 μm in the magnified image in (**C**). See S1 Table for description of quantification and experimental *n*. Raw data can be found in S1 Data.
(TIF)

S2 Fig Whole mount in situ hybridization for zebrafish Lef1-dependent genes identified from RNA-seq. (**A**) Representative images of whole mount in situ hybridization on 3 dpf control and *lef1* mutant embryos. Red and yellow arrows indicate gene expression in caudal and rostral hypothalamus, respectively. Lateral (*adarb2*, *ccdc129*, *foxb2*, *klf17*, *mmp17b*, and *slc18a2*) or ventral (other genes) views were selected for optimal expression visualization. (**B**) Quantification of expression following whole mount in situ hybridization on 3 dpf offspring from *lef1+/-* incrosses. Fifty to eighty-five embryos were analyzed per gene. (**C**) Images of 3 dpf control brains centered on Hc from ventral view. (**D**) Gene expression in the hypothalamus of 4 months post-fertilization (mpf) female wild-type zebrafish from ventral view. Representative images are shown in (**C**) and (**D**) for at least 2 samples tested. Images of ventral view have anterior on top; images of lateral view have dorsal on top and anterior on the left. Red dashed outlines in (**C**) and (**D**) depict the caudal hypothalamus. Scale bars: 0.1 mm in (**A**); 5 μm in (**C**); 0.2 mm in (**D**). Raw data can be found in S1 Data.
(TIF)

S3 Fig Physiological and behavioral analysis of zebrafish *lef1* mutants. (**A**-**C**) Body size and survival rate of *lef1* mutants under different culture conditions. Offspring of *lef1+/-* incrosses were either unsorted or sorted by genotype at 15 dpf, and raised at 25 fish per tank. Body length and number of surviving fish at 2 mpf are shown in (**C**) with representative pictures in (**A**) and (**B**) (*lef1* mutants have no caudal fins [4]). (**D**) Body length of wild-type fish with different culture densities [84]. Data are mean ± SEM. Raw data can be found in S1 Data.
(TIF)

S4 Fig Mouse anxiety tests. (**A**) Elevated plus maze. (**B**) Open field test. In (**A**) and (**B**), *n* **=** 12, 9 for male CON, CKO. In (A), *n* = 11, 11 for female CON, CKO in estrus; *n* = 12, 11 for female CON, CKO in diestrus. In (B), *n* **=** 12, 6 for female CON, CKO in estrus; *n* **=** 11, 16 for female CON, CKO in diestrus. Data are mean ± SEM. **P < 0.01, ns. *P* > 0.05 by unpaired Student *t* tests. Outliers depicted in black (**B**) were excluded from statistical analysis using the Grubbs’ test (*P* < 0.05). Raw data can be found in S1 Data.
(TIF)

S5 Fig Cellular and molecular phenotypes in mouse *Lef1^CKO^* hypothalamus. (**A**) P50 female *Nkx2-1^Cre/+^;Lef1^flox/+^;Rosa^tdTomato/+^* (CON-F) expresses tdTomato in the hypothalamus. Bright field (left) and red fluorescence (right) ventral view images of the same brain with anterior on top are shown. Representative images are shown for at least 3 adult brains dissected. (**B**) Immunostaining for Lef1 in the hypothalamus of E14.5 *Lef1^CON^* (CON) and *Lef1^CKO^* (CKO). Coronal images are z-projections of 16 μm confocal optical sections, shown with dorsal side on top. Representative images are shown for at least 2 replicates tested. (**C**) Immunostaining for Wnt reporter *TCF/Lef*:*H2B-GFP*. Hypothalamic green fluorescence (below) views of yellow rectangles in bright field (above) view images of the same brain are shown, respectively. Images are whole mount ventral views with anterior side on top, acquired with the same setting for CON and CKO. Representative images are shown for at least 3 replicates tested. (**D**) Immunostaining for Pmch in the E14.5 hypothalamus. Higher magnification views of yellow rectangles are shown in the insets. Coronal images are z-projections of 16 μm confocal optical slices, shown with dorsal side on top. (**E**) qPCR analysis for female shows hypothalamic gene expression in E14.5 and P22 CKO-F relative to CON-F. Data are mean ± SEM. ***P < 0.001, ns. *P* > 0.05 by unpaired Student *t* tests. (**F**) Twenty-five μm coronal section in situ hybridization for *Crhbp* in the male P22 ventral premammillary and posterior hypothalamus, shown with dorsal side on top. Representative images are shown for at least 2 replicates tested. 3V: third ventricle. All scale bars are 100 μm except 500 μm in (**F**). Raw data can be found in S1 Data.
(TIF)

S6 Fig Normal expression of *pmch* and *pmchl* in zebrafish *lef1* mutants. (**A**-**C**) Whole mount in situ hybridization images for *pmch* and *pmchl* (*pmch*, *like*) in the hypothalamus of 3 dpf (**A** and **B**) and 15 dpf (**C**) zebrafish control and *lef1* mutant embryos. Images of dorsal views (anterior on top) and lateral views (dorsal on top and anterior on the left) of the same individual *lef1+/-* or *lef1-/-* fish were shown in (**A**). Representative ventral view images of 3 dpf *lef1+/-* (**B**), 15 dpf control and *lef1* mutant (**C**) brains centered on the caudal hypothalamus (dashed red outlines) with anterior on top. Number of fish with representative gene expression among total number of fish is labeled on the right upper corner of each image in (**C**). Scale bar: 100 μm in (**A** and **C**); 5 μm in (**B**).
(TIF)

S1 Table Details of confocal images, quantification and number of samples. (TIF)

S2 Table Zebrafish RNA-seq at 3 dpf. (XLSX)

S3 Table Ingenuity Pathway Analysis (IPA) for diseases & functions. (XLSX)

S4 Table IPA input gene list. Same zebrafish genes in Tab “AdjP<0.1” of S2 Table are listed with orthologous mouse gene information used for IPA.
(XLSX)

S5 Table Mouse RNA-seq at E14.5. (XLSX)

S6 Table Mouse RNA-seq at P22. (XLSX)

S7 Table Extracted GTEx RNA-seq data from 96 human hypothalamic samples. (ZIP)

S8 Table Pearson correlation with hypothalamic GTEx RNA-seq data for *PMCH*, *CRHRP*, *LEF1* and *NPY*. Gene name followed with “_r” indicates Pearson’s *r* value and gene name followed with “_p” indicates *P* value.
(ZIP)

S9 Table Primer sequences for synthesizing in situ hybridization probes. Reverse primers also included a T7 promoter-containing sequence “CCAAGCTTCTAATACGACTCACTATAGGGAGA” that was added 5' to the sequences listed in the table [70]. All primers were designed by Primer-BLAST except mouse genes *Cartpt* (ABA experiment 72077479), *Crhbp* (ABA experiment 77455017), *Pmch* (GenePaint set MH227) and *Tacr3* (ABA experiment 80342167).
(XLSX)

S1 Data Excel spreadsheet containing, in separate sheets, the underlying numerical data for all the figure panels. (XLSX)

S1 Video One representative video of novel tank diving test. The resolution of the video was reduced from original 1080p to 540p, and its dimension was cropped to remove unnecessary space using software HandBrake.
(MP4)

## Abbreviations

AdjP: adjusted *P* value
AGRP: agouti-related protein
Cartpt: CART prepropeptide
CRH: corticotropin-releasing hormone
dpf: days post-fertilization
CRHBP: corticotropin-releasing hormone binding protein
E: embryonic day
EPM: elevated plus maze
GTEx: Genotype-Tissue Expression
Hc: caudal hypothalamus
HPA: hypothalamic-pituitary-adrenal
IPA: Ingenuity Pathway Analysis
NPY: neuropeptide Y
OFT: open field test
P: postnatal day
pan: pangolin
PI: pars intercerebralis
PL: pars lateralis
PMCH: pro-melanin concentrating hormone
qPCR: quantitative real-time PCR
RNA-seq: RNA sequencing
RPKM: reads per kilobase of transcript per million mapped reads
Tacr3: tachykinin receptor 3
wt: wild-type

## References

[1] Mohammad F, Aryal S, Ho J, Stewart JC, Norman NA, Tan TL, et al Ancient anxiety pathways influence Drosophila defense behaviors. Curr Biol. 2016;26(7):981–6. doi: 10.1016/j.cub.2016.02.031 27020741 10.1016/j.cub.2016.02.031 PMC4826436

[2] Xie Y, Dorsky RI. Development of the hypothalamus: conservation, modification and innovation. Development. 2017;144(9):1588–99. doi: 10.1242/dev.139055 28465334 10.1242/dev.139055 PMC5450842

[3] Nusse R, Clevers H. Wnt/β-catenin signaling, disease, and emerging therapeutic modalities. Cell. 2017 6 1;169(6):985–99. doi: 10.1016/j.cell.2017.05.016 28575679 10.1016/j.cell.2017.05.016

[4] Wang X, Kopinke D, Lin J, McPherson AD, Duncan RN, Otsuna H, et al Wnt signaling regulates postembryonic hypothalamic progenitor differentiation. Dev Cell. 2012 9 11;23(3):624–36. doi: 10.1016/j.devcel.2012.07.012 22975330 10.1016/j.devcel.2012.07.012 PMC3445042

[5] Löhr H, Hammerschmidt M. Zebrafish in endocrine systems: recent advances and implications for human disease. Annu Rev Physiol. 2011;73:183–211. doi: 10.1146/annurev-physiol-012110-142320 21314433 10.1146/annurev-physiol-012110-142320

[6] Lonsdale J, Thomas J, Salvatore M, Phillips R, Lo E, Shad S, et al The Genotype-Tissue Expression (GTEx) project. Nat Genet. 2013;45(6):580–5. doi: 10.1038/ng.2653 23715323 10.1038/ng.2653 PMC4010069

[7] Dorsky RI, Sheldahl LC, Moon RT. A transgenic Lef1/beta-catenin-dependent reporter is expressed in spatially restricted domains throughout zebrafish development. Dev Biol. 2002 1 15;241(2):229–37. doi: 10.1006/dbio.2001.0515 11784107 10.1006/dbio.2001.0515

[8] McPherson AD, Barrios JP, Luks-Morgan SJ, Manfredi JP, Bonkowsky JL, Douglass AD, et al Motor behavior mediated by continuously generated dopaminergic neurons in the zebrafish hypothalamus recovers after cell ablation. Curr Biol. 2015;26(2):263–9. doi: 10.1016/j.cub.2015.11.064 26774784 10.1016/j.cub.2015.11.064 PMC4864152

[9] Duncan RN, Xie Y, McPherson AD, Taibi A V, Bonkowsky JL, Douglass AD, et al Hypothalamic radial glia function as self-renewing neural progenitors in the absence of Wnt/β-catenin signaling. Development. 2016 1 1;143(1):45–53. doi: 10.1242/dev.126813 26603385 10.1242/dev.126813 PMC4725207

[10] MacDonald BT, Tamai K, He X. Wnt/beta-catenin signaling: components, mechanisms, and diseases. Dev Cell. 2009;17(1):9–26. doi: 10.1016/j.devcel.2009.06.016 19619488 10.1016/j.devcel.2009.06.016 PMC2861485

[11] Weidinger G, Thorpe CJ, Wuennenberg-Stapleton K, Ngai J, Moon RT. The Sp1-related transcription factors sp5 and sp5-like act downstream of Wnt/beta-catenin signaling in mesoderm and neuroectoderm patterning. Curr Biol. 2005 3 29;15(6):489–500. doi: 10.1016/j.cub.2005.01.041 15797017 10.1016/j.cub.2005.01.041

[12] Carr JA. Stress, neuropeptides, and feeding behavior: a comparative perspective. Integr Comp Biol. 2002;42(3):582–90. doi: 10.1093/icb/42.3.582 21708754 10.1093/icb/42.3.582

[13] Cachat JM, Canavello PR, Elkhayat SI, Bartels BK, Hart PC, Elegante MF, et al. Video-aided analysis of zebrafish locomotion and anxiety-related behavioral responses. In: Zebrafish Neurobehavioral Protocols. 2011. p. 1–14.doi: 10.1007/978-1-60761-953-6_1 27019459

[14] Karolyi IJ, Burrows HL, Ramesh TM, Nakajima M, Lesh JS, Seong E, et al Altered anxiety and weight gain in corticotropin-releasing hormone-binding protein-deficient mice. Proc Natl Acad Sci U S A. 1999;96(9):11595–600. 10500222 10.1073/pnas.96.20.11595 PMC18079

[15] Mcgraw HF, Drerup CM, Culbertson MD, Linbo T, Raible DW, Nechiporuk A V. Lef1 is required for progenitor cell identity in the zebrafish lateral line primordium. Development. 2011 9;138(18):3921–30. doi: 10.1242/dev.062554 21862556 10.1242/dev.062554 PMC3160089

[16] Ng L, Bernard A, Lau C, Overly CC, Dong H-W, Kuan C, et al An anatomic gene expression atlas of the adult mouse brain. Nat Neurosci. 2009;12(3):356–62. doi: 10.1038/nn.2281 19219037 10.1038/nn.2281

[17] Shimogori T, Lee DA, Miranda-Angulo A, Yang Y, Wang H, Jiang L, et al A genomic atlas of mouse hypothalamic development. Nat Neurosci. 2010 7;13(6):767–75. doi: 10.1038/nn.2545 20436479 10.1038/nn.2545 PMC4067769

[18] van Genderen C, Okamura RM, Fariñas I, Quo RG, Parslow TG, Bruhn L, et al Development of several organs that require inductive epithelial-mesenchymal interactions is impaired in LEF-1-deficient mice. Genes Dev. 1994 11 15;8(22):2691–703. 7958926 10.1101/gad.8.22.2691

[19] Galceran J, Miyashita-Lin EM, Devaney E, Rubenstein JL, Grosschedl R. Hippocampus development and generation of dentate gyrus granule cells is regulated by LEF1. Development. 2000 2;127(3):469–82. 10631168 10.1242/dev.127.3.469

[20] Yu S, Zhou X, Steinke FC, Liu C, Chen S, Zagorodna O, et al The TCF-1 and LEF-1 transcription factors have cooperative and opposing roles in T cell development and malignancy. Immunity. 2012 11 16;37(5):1–26. doi: 10.1016/j.immuni.2012.08.009 23103132 10.1016/j.immuni.2012.08.009 PMC3501598

[21] Xu Q, Tam M, Anderson SA. Fate mapping Nkx2.1-lineage cells in the mouse telencephalon. J Comp Neurol. 2008;29(9 2007):16–29. doi: 10.1002/cne.21529 17990269 10.1002/cne.21529

[22] Madisen L, Zwingman TA, Sunkin SM, Oh SW, Zariwala HA, Gu H, et al A robust and high-throughput Cre reporting and characterization system for the whole mouse brain. Nat Neurosci. 2010 1;13(1):133–40. doi: 10.1038/nn.2467 20023653 10.1038/nn.2467 PMC2840225

[23] Ferrer-Vaquer A, Piliszek A, Tian G, Aho RJ, Dufort D, Hadjantonakis A-K. A sensitive and bright single-cell resolution live imaging reporter of Wnt/ß-catenin signaling in the mouse. BMC Dev Biol. 2010;10:121 doi: 10.1186/1471-213X-10-121 21176145 10.1186/1471-213X-10-121 PMC3017038

[24] Bale TL, Epperson CN. Sex differences and stress across the lifespan. Nat Neurosci. 2015;18(10):35–42. doi: 10.1038/nn.4112 26404716 10.1038/nn.4112 PMC4620712

[25] Clément Y, Calatayud F, Belzung C. Genetic basis of anxiety-like behaviour: A critical review. Brain Res Bull. 2002;57(1):57–71. 11827738 10.1016/s0361-9230(01)00637-2

[26] Visel A, Thaller C, Eichele G. GenePaint.org: an atlas of gene expression patterns in the mouse embryo. Nucleic Acids Res. 2004;32(Database issue):D552–6. doi: 10.1093/nar/gkh029 14681479 10.1093/nar/gkh029 PMC308763

[27] Brischoux F, Fellmann D, Risold PY. Ontogenetic development of the diencephalic MCH neurons: A hypothalamic “MCH area” hypothesis. Eur J Neurosci. 2001;13(9):1733–44. 11359525 10.1046/j.0953-816x.2001.01552.x

[28] Alon T, Friedman JM. Late-onset leanness in mice with targeted ablation of melanin concentrating hormone neuron. J Neurosci. 2006;26(2):389–97. doi: 10.1523/JNEUROSCI.1203-05.2006 16407534 10.1523/JNEUROSCI.1203-05.2006 PMC6674397

[29] Whiddon BB, Palmiter RD. Ablation of neurons expressing melanin-concentrating hormone (MCH) in adult mice improves glucose tolerance independent of MCH signaling. J Neurosci. 2013;33(5):2009–16. doi: 10.1523/JNEUROSCI.3921-12.2013 23365238 10.1523/JNEUROSCI.3921-12.2013 PMC3725743

[30] González JA, Iordanidou P, Strom M, Adamantidis A, Burdakov D. Awake dynamics and brain-wide direct inputs of hypothalamic MCH and orexin networks. Nat Commun. 2016;7(c):11395 doi: 10.1038/ncomms11395 27102565 10.1038/ncomms11395 PMC4844703

[31] Berman JR, Skariah G, Maro GS, Mignot E, Mourrain P. Characterization of two melanin-concentrating hormone genes in zebrafish reveals evolutionary and physiological links with the mammalian MCH system. J Comp Neurol. 2009;517(5):695–710. doi: 10.1002/cne.22171 19827161 10.1002/cne.22171 PMC3427774

[32] Hartenstein V. The neuroendocrine system of invertebrates: a developmental and evolutionary perspective. J Endocrinol. 2006;190(3):555–70. doi: 10.1677/joe.1.06964 17003257 10.1677/joe.1.06964

[33] Brunner E, Peter O, Schweizer L, Basler K. pangolin encodes a Lef-1 homologue that acts downstream of Armadillo to transduce the Wingless signal in Drosophila. Vol. 385, Nature. 1997 p. 829–33. doi: 10.1038/385829a0 9039917 10.1038/385829a0

[34] Van de Wetering M, Cavallo R, Dooijes D, Van Beest M, Van Es J, Loureiro J, et al Armadillo coactivates transcription driven by the product of the Drosophila segment polarity gene dTCF. Cell. 1997;88(6):789–99. 9118222 10.1016/s0092-8674(00)81925-x

[35] Huising MO, Flik G. The remarkable conservation of corticotropin-releasing hormone (CRH)-binding protein in the honeybee (Apis mellifera) dates the CRH system to a common ancestor of insects and vertebrates. Endocrinology. 2005;146(5):2165–70. doi: 10.1210/en.2004-1514 15718273 10.1210/en.2004-1514

[36] Croizier S, Cardot J, Brischoux F, Fellmann D, Griffond B, Risold PY. The vertebrate diencephalic MCH system: A versatile neuronal population in an evolving brain. Front Neuroendocrinol. 2013;34(2):65–87. doi: 10.1016/j.yfrne.2012.10.001 23088995 10.1016/j.yfrne.2012.10.001

[37] Mele M, Ferreira PG, Reverter F, DeLuca DS, Monlong J, Sammeth M, et al The human transcriptome across tissues and individuals. Science (80-). 2015 5 7;348(6235):660–5. doi: 10.1126/science.aaa0355 25954002 10.1126/science.aaa0355 PMC4547472

[38] Böhm J, Sustmann C, Wilhelm C, Kohlhase J, Bo J, Sustmann C, et al SALL4 is directly activated by TCF/LEF in the canonical Wnt signaling pathway. Biochem Biophys Res Commun. 2006 9 29;348(3):898–907. doi: 10.1016/j.bbrc.2006.07.124 16899215 10.1016/j.bbrc.2006.07.124

[39] Ardlie KG, Deluca DS, Segre A V., Sullivan TJ, Young TR, Gelfand ET, et al The Genotype-Tissue Expression (GTEx) pilot analysis: Multitissue gene regulation in humans. Science (80-). 2015 5 7;348(6235):648–60. doi: 10.1126/science.1262110 25954001 10.1126/science.1262110 PMC4547484

[40] Jin Y-R, Yoon JK. The R-spondin family of proteins: emerging regulators of WNT signaling. Int J Biochem Cell Biol. 2012 12;44(12):2278–87. doi: 10.1016/j.biocel.2012.09.006 22982762 10.1016/j.biocel.2012.09.006 PMC3496018

[41] Schwartz MW, Hahn TM, Breininger JF, Baskin DG. Coexpression of Agrp and NPY in fasting-activated hypothalamic neurons. Nat Neurosci. 1998 8 1;1(4):271–2. doi: 10.1038/1082 10195157 10.1038/1082

[42] Mulligan MK, Mozhui K, Prins P, Williams RW. GeneNetwork: A Toolbox for Systems Genetics. In: Methods Mol Biol. 2017 p. 75–120. doi: 10.1007/978-1-4939-6427-7_4 27933521 10.1007/978-1-4939-6427-7_4 PMC7495243

[43] Kaslin J, Panula P. Comparative anatomy of the histaminergic and other aminergic systems in zebrafish (Danio rerio). J Comp Neurol. 2001;440(4):342–77. 11745628 10.1002/cne.1390

[44] Wullimann MF, Rupp B, Reichert H. Neuroanatomy of the zebrafish brain: A topological atlas Birkhäuser Verlag Basel: Birkhäuser Basel; 1996. 144 p.

[45] Baker BI. Melanin-concentrating hormone: a general vertebrate neuropeptide. Int Rev Cytol. 1991;126:1–47. 2050497 10.1016/s0074-7696(08)60681-6

[46] Griffond B, Baker BI. Cell and molecular cell biology of melanin-concentrating hormone. Vol. 213, Int. Rev. Cytol. Elsevier Masson SAS; 2002 233–277 p. 11837894 10.1016/s0074-7696(02)13016-6

[47] Chung S, Parks GS, Lee C, Civelli O. Recent updates on the melanin-concentrating hormone (MCH) and its receptor system: Lessons from MCH1R antagonists. J Mol Neurosci. 2011;43(1):115–21. doi: 10.1007/s12031-010-9411-4 20582487 10.1007/s12031-010-9411-4 PMC3018593

[48] Bittencourt J, Celis ME. Anatomy, function and regulation of neuropeptide EI (NEI). Peptides. 2008;29(8):1441–50. doi: 10.1016/j.peptides.2008.03.012 18456371 10.1016/j.peptides.2008.03.012

[49] Shimada M, Tritos NA, Lowell BB, Flier JS, Maratos-Flier E. Mice lacking melanin-concentrating hormone are hypophagic and lean. Nature. 1998;396(12):670–4. doi: 10.1038/25341 9872314 10.1038/25341

[50] Kokkotou E, Jeon JY, Wang X, Marino FE, Carlson M, Trombly DJ, et al Mice with MCH ablation resist diet-induced obesity through strain-specific mechanisms. Am J Physiol Regul Integr Comp Physiol. 2005;289:R117–24. doi: 10.1152/ajpregu.00861.2004 15731402 10.1152/ajpregu.00861.2004

[51] Zhou D, Shen Z, Strack AM, Marsh DJ, Shearman LP. Enhanced running wheel activity of both Mch1r- and Pmch-deficient mice. Regul Pept. 2005;124(1–3):53–63. doi: 10.1016/j.regpep.2004.06.026 15544841 10.1016/j.regpep.2004.06.026

[52] Willie JT, Sinton CM, Maratos-Flier E, Yanagisawa M. Abnormal response of melanin-concentrating hormone deficient mice to fasting: Hyperactivity and rapid eye movement sleep suppression. Neuroscience. 2008;156(4):819–29. doi: 10.1016/j.neuroscience.2008.08.048 18809470 10.1016/j.neuroscience.2008.08.048 PMC2586720

[53] Mul JD, Yi C-X, van den Berg SAA, Ruiter M, Toonen PW, van der Elst MCJ, et al Pmch expression during early development is critical for normal energy homeostasis. Am J Physiol Endocrinol Metab. 2010;298(3):E477–88. doi: 10.1152/ajpendo.00154.2009 19934402 10.1152/ajpendo.00154.2009

[54] Nocedal I, Johnson AD. How transcription networks evolve and produce biological novelty. Cold Spring Harb Symp Quant Biol. 2015;LXXX doi: 10.1101/sqb.2015.80.027557 26657905 10.1101/sqb.2015.80.027557

[55] Villar D, Flicek P, Odom DT. Evolution of transcription factor binding in metazoans—mechanisms and functional implications. Nat Rev Genet. 2014 4;15(4):221–33. doi: 10.1038/nrg3481 24590227 10.1038/nrg3481 PMC4175440

[56] Sokolowski K, Esumi S, Hirata T, Kamal Y, Tran T, Lam A, et al Specification of select hypothalamic circuits and innate behaviors by the embryonic patterning gene Dbx1. Neuron. 2015;86(2):1–14. doi: 10.1016/j.neuron.2015.03.022 25864637 10.1016/j.neuron.2015.03.022 PMC4484744

[57] Duman RS, Voleti B. Signaling pathways underlying the pathophysiology and treatment of depression: Novel mechanisms for rapid-acting agents. Trends Neurosci. 2012;35(1):47–56. doi: 10.1016/j.tins.2011.11.004 22217452 10.1016/j.tins.2011.11.004 PMC3278537

[58] Dias C, Feng J, Sun H, Shao NY, Mazei-Robison MS, Damez-Werno D, et al β-catenin mediates stress resilience through Dicer1/microRNA regulation. Nature. 2014 11 12;516(7529):51–5. doi: 10.1038/nature13976 25383518 10.1038/nature13976 PMC4257892

[59] Lee JE, Wu S, Goering LM, Dorsky RI. Canonical Wnt signaling through Lef1 is required for hypothalamic neurogenesis. Development. 2006 11;133(22):4451–61. doi: 10.1242/dev.02613 17050627 10.1242/dev.02613

[60] Ghanem N, Jarinova O, Amores A, Long Q, Hatch G, Park BK, et al Regulatory roles of conserved intergenic domains in vertebrate Dlx bigene clusters. Genome Res. 2003 4;13(4):533–43. doi: 10.1101/gr.716103 12670995 10.1101/gr.716103 PMC430168

[61] Pauls S, Geldmacher-Voss B, Campos-Ortega JA. A zebrafish histone variant H2A.F/Z and a transgenic H2A.F/Z:GFP fusion protein for in vivo studies of embryonic development. Dev Genes Evol. 2001 12;211(12):603–10. doi: 10.1007/s00427-001-0196-x 11819118 10.1007/s00427-001-0196-x

[62] Berghmans S, Murphey RD, Wienholds E, Neuberg D, Kutok JL, Fletcher CDM, et al tp53 mutant zebrafish develop malignant peripheral nerve sheath tumors. Proc Natl Acad Sci U S A. 2005 1 11;102(2):407–12. doi: 10.1073/pnas.0406252102 15630097 10.1073/pnas.0406252102 PMC544293

[63] Johnson CW, Hernandez-Lagunas L, Feng W, Melvin VS, Williams T, Artinger KB. Vgll2a is required for neural crest cell survival during zebrafish craniofacial development. Dev Biol. 2011 9;357(1):269–81. doi: 10.1016/j.ydbio.2011.06.034 21741961 10.1016/j.ydbio.2011.06.034 PMC3519931

[64] Gil-Sanz C, Espinosa A, Fregoso SP, Bluske KK, Cunningham CL, Martinez-Garay I, et al Lineage tracing using Cux2-Cre and Cux2-CreERT2 mice. Neuron. 2015;86(4):1091–9. doi: 10.1016/j.neuron.2015.04.019 25996136 10.1016/j.neuron.2015.04.019 PMC4455040

[65] Clapcote SJ, Roder JC. Simplex PCR assay for sex determination in mice. Biotechniques. 2005;38(5):702–6. 15945368 10.2144/05385BM05

[66] Woo K, Fraser SE. Order and coherence in the fate map of the zebrafish nervous system. Development. 1995 8;121(8):2595–609. 7671822 10.1242/dev.121.8.2595

[67] Eisenhoffer GT, Loftus PD, Yoshigi M, Otsuna H, Chien C-B, Morcos PA, et al Crowding induces live cell extrusion to maintain homeostatic cell numbers in epithelia. Nature. 2012 4 15;484(7395):546–9. doi: 10.1038/nature10999 22504183 10.1038/nature10999 PMC4593481

[68] Byars CL, Bates KL, Letsou A. The dorsal-open group gene raw is required for restricted DJNK signaling during closure. Development. 1999;126:4913–23. 10518507 10.1242/dev.126.21.4913

[69] Thisse C, Thisse B. High-resolution in situ hybridization to whole-mount zebrafish embryos. Nat Protoc. 2008 1;3(1):59–69. doi: 10.1038/nprot.2007.514 18193022 10.1038/nprot.2007.514

[70] Logel J, Dill D, Leonard S. Synthesis of cRNA probes from PCR-generated DNA. Biotechniques. 1992;13(4):604–606+608. 1476730

[71] Ye J, Coulouris G, Zaretskaya I, Cutcutache I, Rozen S, Madden TL. Primer-BLAST: a tool to design target-specific primers for polymerase chain reaction. BMC Bioinformatics. 2012 1;13:134 doi: 10.1186/1471-2105-13-134 22708584 10.1186/1471-2105-13-134 PMC3412702

[72] Dorsky RI, Snyder A, Cretekos CJ, Grunwald DJ, Geisler R, Haffter P, et al Maternal and embryonic expression of zebrafish lef1. Mech Dev. 1999;86(1–2):147–50. 10446273 10.1016/s0925-4773(99)00101-x

[73] Wang X, Lee JE, Dorsky RI. Identification of Wnt-responsive cells in the zebrafish hypothalamus. Zebrafish. 2009;6(1):49–58. doi: 10.1089/zeb.2008.0570 19374548 10.1089/zeb.2008.0570 PMC2765247

[74] Stapleton M, Carlson J, Brokstein P, Yu C, Champe M, George R, et al A Drosophila full-length cDNA resource. Genome Biol. 2002;3(12):RESEARCH0080 doi: 10.1186/gb-2002-3-12-research0080 12537569 10.1186/gb-2002-3-12-research0080 PMC151182

[75] Oxtoby E, Jowett T. Cloning of the zebrafish krox-20 gene (krx-20) and its expression during hindbrain development. Nucleic Acids Res. 1993;21(5):1087–95. 8464695 10.1093/nar/21.5.1087 PMC309267

[76] Thompson CL, Ng L, Menon V, Martinez S, Lee CK, Glattfelder K, et al A high-resolution spatiotemporal atlas of gene expression of the developing mouse brain. Neuron. 2014;83(2):309–23. doi: 10.1016/j.neuron.2014.05.033 24952961 10.1016/j.neuron.2014.05.033 PMC4319559

[77] Schaeren-Wiemers N, Gerfin-Moser A. A single protocol to detect transcripts of various types and expression levels in neural tissue and cultured cells: in situ hybridization using digoxigenin-labelled cRNA probes. Histochemistry. 1993 12;100(6):431–40. 7512949 10.1007/BF00267823

[78] Hurst JL, West RS. Taming anxiety in laboratory mice. Nat Methods. 2010;7(10):825–6. doi: 10.1038/nmeth.1500 20835246 10.1038/nmeth.1500

[79] Hånell A, Marklund N. Structured evaluation of rodent behavioral tests used in drug discovery research. Front Behav Neurosci. 2014;8(7):252 doi: 10.3389/fnbeh.2014.00252 25100962 10.3389/fnbeh.2014.00252 PMC4106406

[80] McLean AC, Valenzuela N, Fai S, Bennett SAL. Performing vaginal lavage, crystal violet staining, and vaginal cytological evaluation for mouse estrous cycle staging identification. J Vis Exp. 2012;(67):e4389 doi: 10.3791/4389 23007862 10.3791/4389 PMC3490233

[81] Schmittgen TD, Livak KJ. Analyzing real-time PCR data by the comparative CT method. Nat Protoc. 2008 6;3(6):1101–8. 18546601 10.1038/nprot.2008.73

[82] Spandidos A, Wang X, Wang H, Seed B. PrimerBank: a resource of human and mouse PCR primer pairs for gene expression detection and quantification. Nucleic Acids Res. 2010 1;38(Database issue):D792–9. doi: 10.1093/nar/gkp1005 19906719 10.1093/nar/gkp1005 PMC2808898

[83] Yu M, Xi Y, Pollack J, Debiais-Thibaud M, MacDonald RB, Ekker M. Activity of dlx5a/dlx6a regulatory elements during zebrafish GABAergic neuron development. Int J Dev Neurosci. 2011;29(7):681–91. doi: 10.1016/j.ijdevneu.2011.06.005 21723936 10.1016/j.ijdevneu.2011.06.005

[84] Reed B, Jennings M. Guidance on the housing and care of Zebrafish Danio rerio. Res Anim Dep Sci Group, RSPCA, West Sussex, United Kingdom 2010.

