# Supplementary figures and images for: Lef1-dependent hypothalamic neurogenesis inhibits anxiety
Source: PLoS Biol. 2017 Aug 24;15(8):e2002257. doi: 10.1371/journal.pbio.2002257 (PMC5570277; doi:10.1371/journal.pbio.2002257)

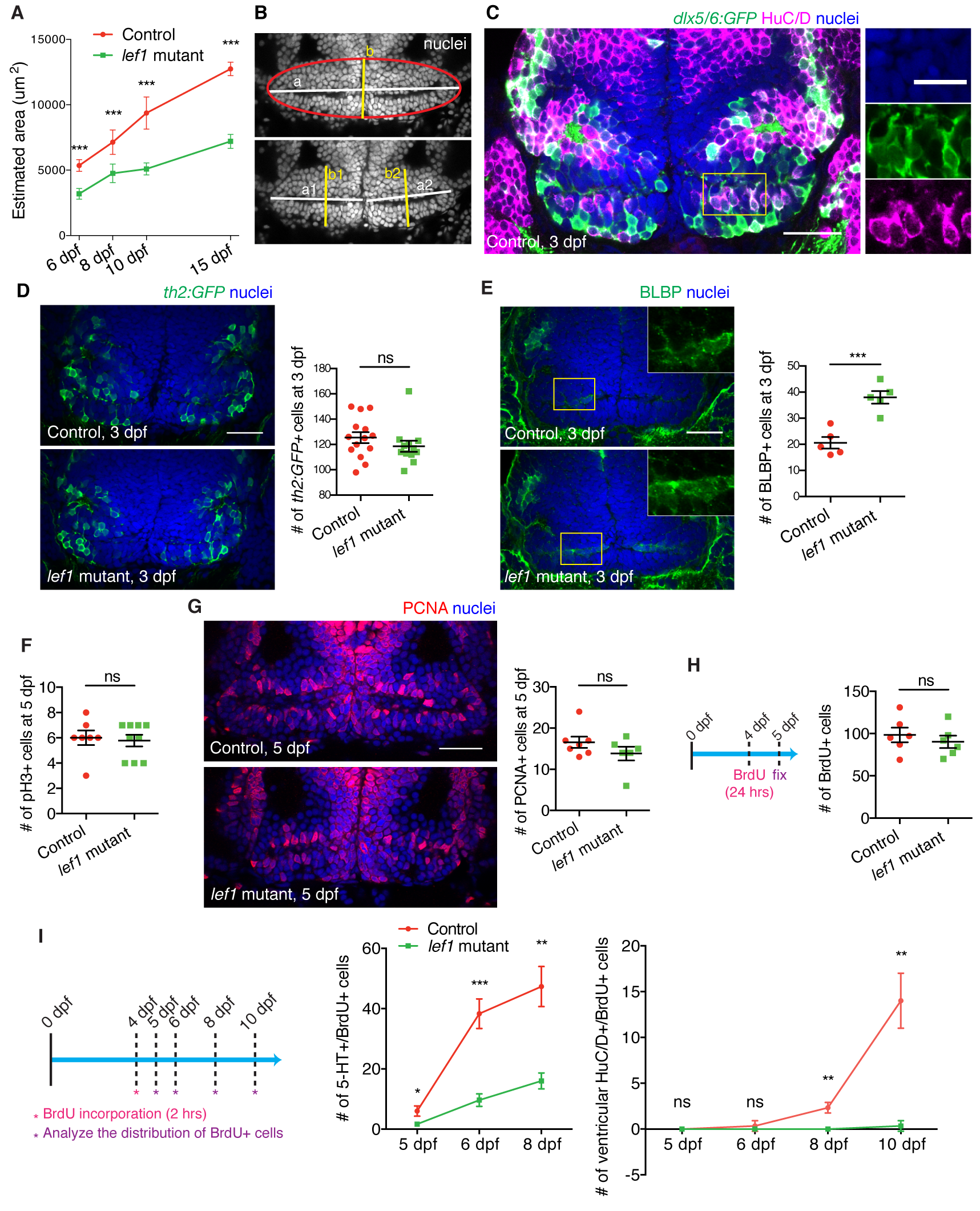

Supplement: S1 Fig — (A and B) Hc size in control and lef1 mutants (A) estimated by the area of confocal ventricular slice (B). Hc was defined as an oval indicated by red outline in (B). The lengths of a1, a2, b1, b2 in the representative image (B) were measured by ImageJ, and the area of the oval was calculated by the following equations: Estimated area = π*a*b/4; a = a1+a2; b = (b1+b2)/2. (C) Co-immunostaining of HuC/D and GABAergic lineage marker dlx5/6:GFP [83] in the 3 dpf Hc. Three confocal channel-split magnified images of the region depicted by the yellow rectangle are shown on the right. A representative image is shown for at least 3 embryos tested. (D and E) Immunostaining of th2:GFP+ (D) and BLBP+ cells (E) in the Hc of 3 dpf control and lef1 mutant. Representative images are shown on the left, and quantifications are shown on the right. Higher magnification views of yellow rectangles in single channel are shown in the insets in (E). (F-H) Measurement of proliferation in the Hc of 5 dpf control and lef1 mutant as shown by pH3+ (F) and PCNA+ cells (G; representative image on the left and quantification on the right; cells adjacent to the horizontal ventricle were counted), and 1 day BrdU labeling (H; schematic on the left). (I), BrdU pulse-chase (schematic on the left) to measure birth of 5-HT+ and ventricular HuC/D+ cells after 4 dpf. Data are mean ± SEM, except mean ± SD in (A) and (I). ***P < 0.001, **P < 0.01, *P < 0.05, ns. P > 0.05 by unpaired Student t tests. All images are confocal ventricular slices. All scale bars are 25 μm except 12.5 μm in the magnified image in (C). See S1 Table for description of quantification and experimental n. Raw data can be found in S1 Data. (TIF) [file pbio.2002257.s001.tif]

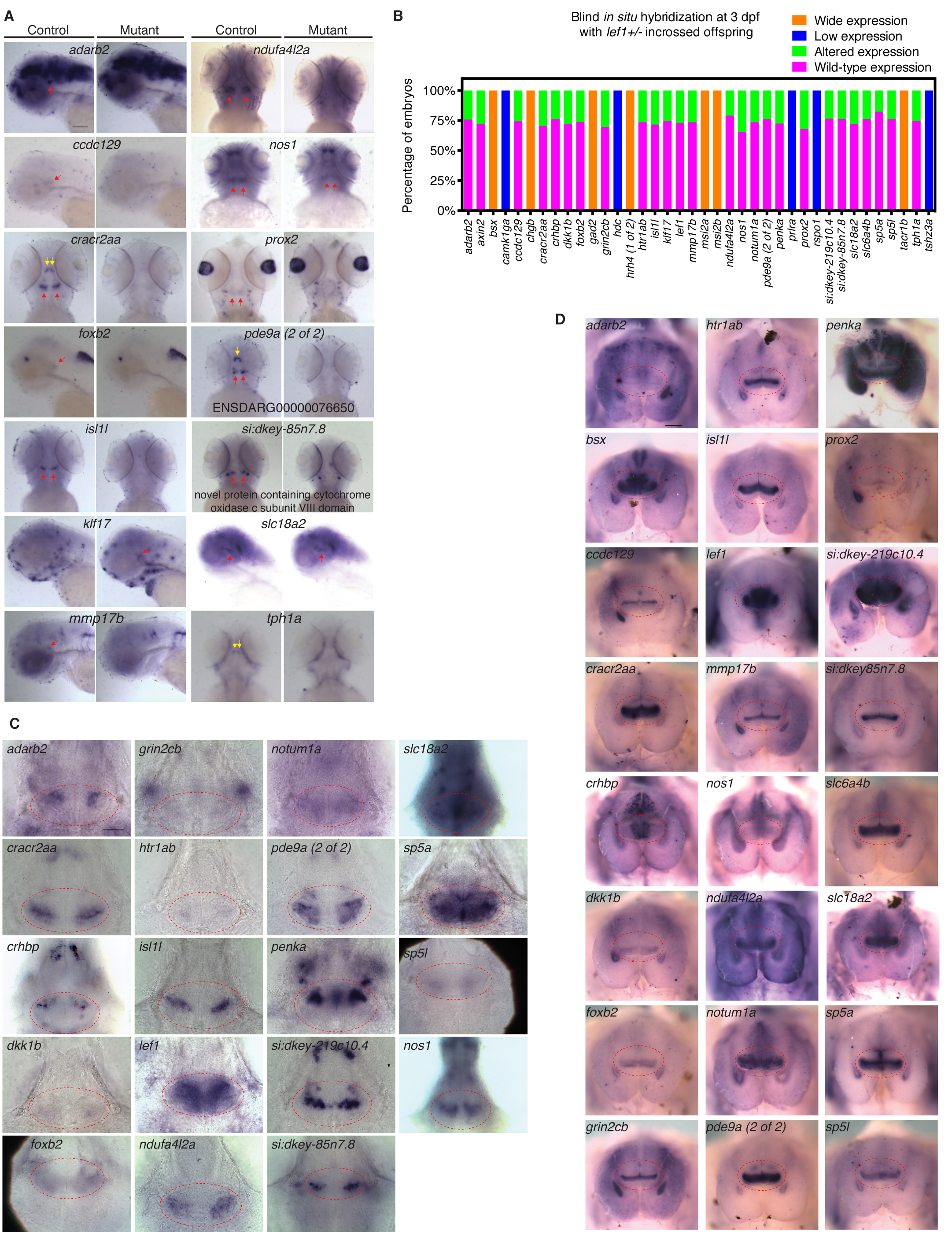

Supplement: S2 Fig — (A) Representative images of whole mount in situ hybridization on 3 dpf control and lef1 mutant embryos. Red and yellow arrows indicate gene expression in caudal and rostral hypothalamus, respectively. Lateral (adarb2, ccdc129, foxb2, klf17, mmp17b, and slc18a2) or ventral (other genes) views were selected for optimal expression visualization. (B) Quantification of expression following whole mount in situ hybridization on 3 dpf offspring from lef1+/- incrosses. Fifty to eighty-five embryos were analyzed per gene. (C) Images of 3 dpf control brains centered on Hc from ventral view. (D) Gene expression in the hypothalamus of 4 months post-fertilization (mpf) female wild-type zebrafish from ventral view. Representative images are shown in (C) and (D) for at least 2 samples tested. Images of ventral view have anterior on top; images of lateral view have dorsal on top and anterior on the left. Red dashed outlines in (C) and (D) depict the caudal hypothalamus. Scale bars: 0.1 mm in (A); 5 μm in (C); 0.2 mm in (D). Raw data can be found in S1 Data. (TIF) [file pbio.2002257.s002.tif]

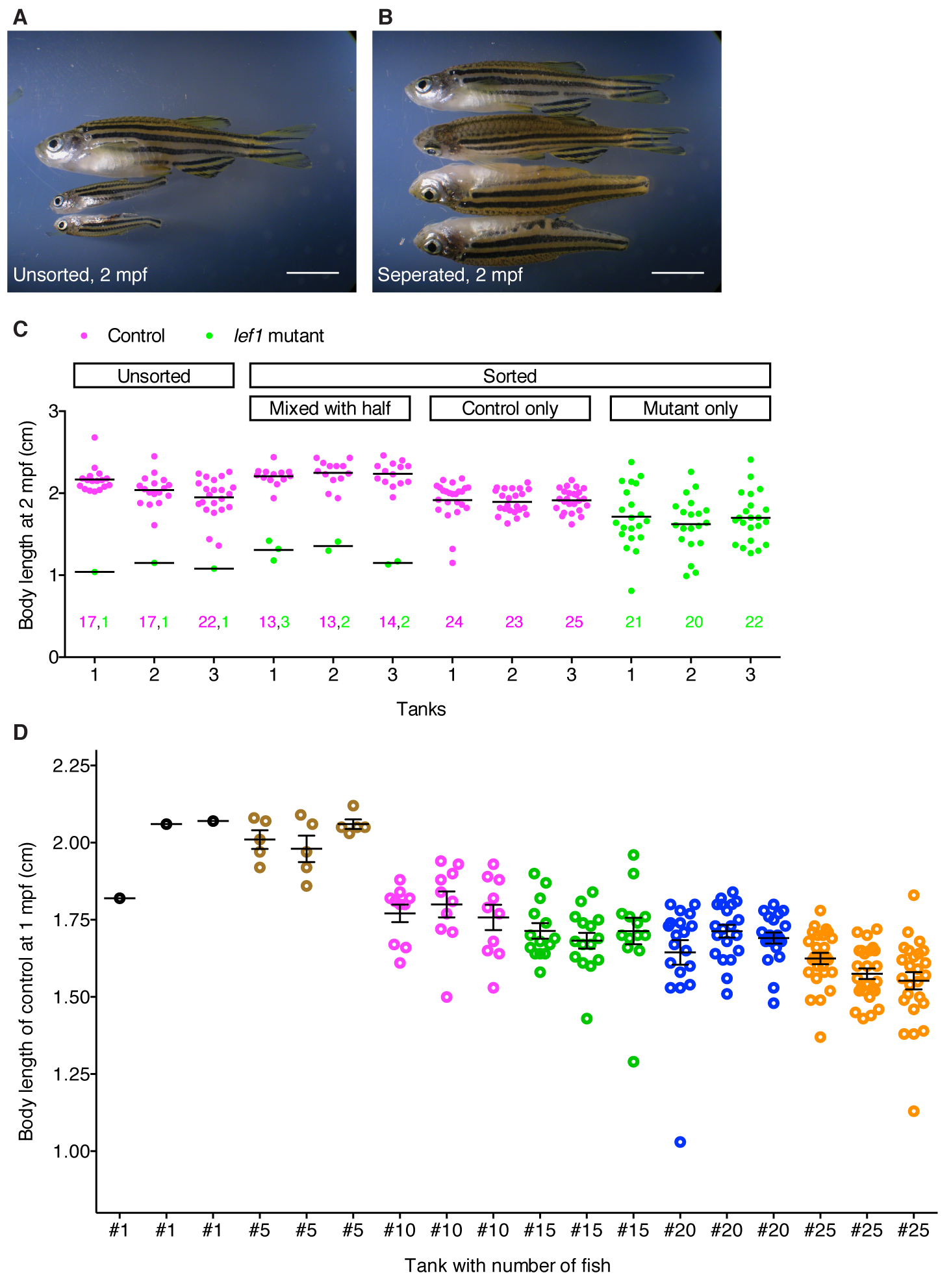

Supplement: S3 Fig — (A-C) Body size and survival rate of lef1 mutants under different culture conditions. Offspring of lef1+/- incrosses were either unsorted or sorted by genotype at 15 dpf, and raised at 25 fish per tank. Body length and number of surviving fish at 2 mpf are shown in (C) with representative pictures in (A) and (B) (lef1 mutants have no caudal fins [4]). (D) Body length of wild-type fish with different culture densities [84]. Data are mean ± SEM. Raw data can be found in S1 Data. (TIF) [file pbio.2002257.s003.tif]

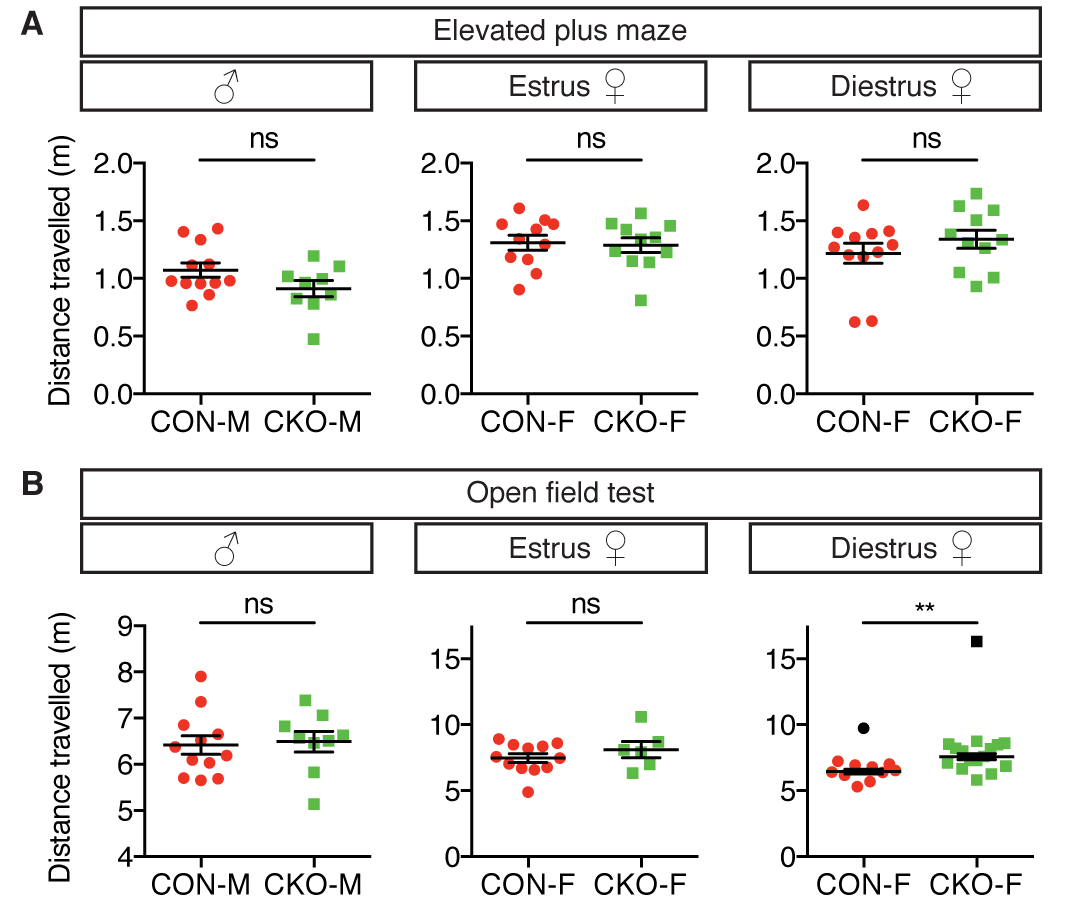

Supplement: S4 Fig — (A) Elevated plus maze. (B) Open field test. In (A) and (B), n = 12, 9 for male CON, CKO. In (A), n = 11, 11 for female CON, CKO in estrus; n = 12, 11 for female CON, CKO in diestrus. In (B), n = 12, 6 for female CON, CKO in estrus; n = 11, 16 for female CON, CKO in diestrus. Data are mean ± SEM. **P < 0.01, ns. P > 0.05 by unpaired Student t tests. Outliers depicted in black (B) were excluded from statistical analysis using the Grubbs’ test (P < 0.05). Raw data can be found in S1 Data. (TIF) [file pbio.2002257.s004.tif]

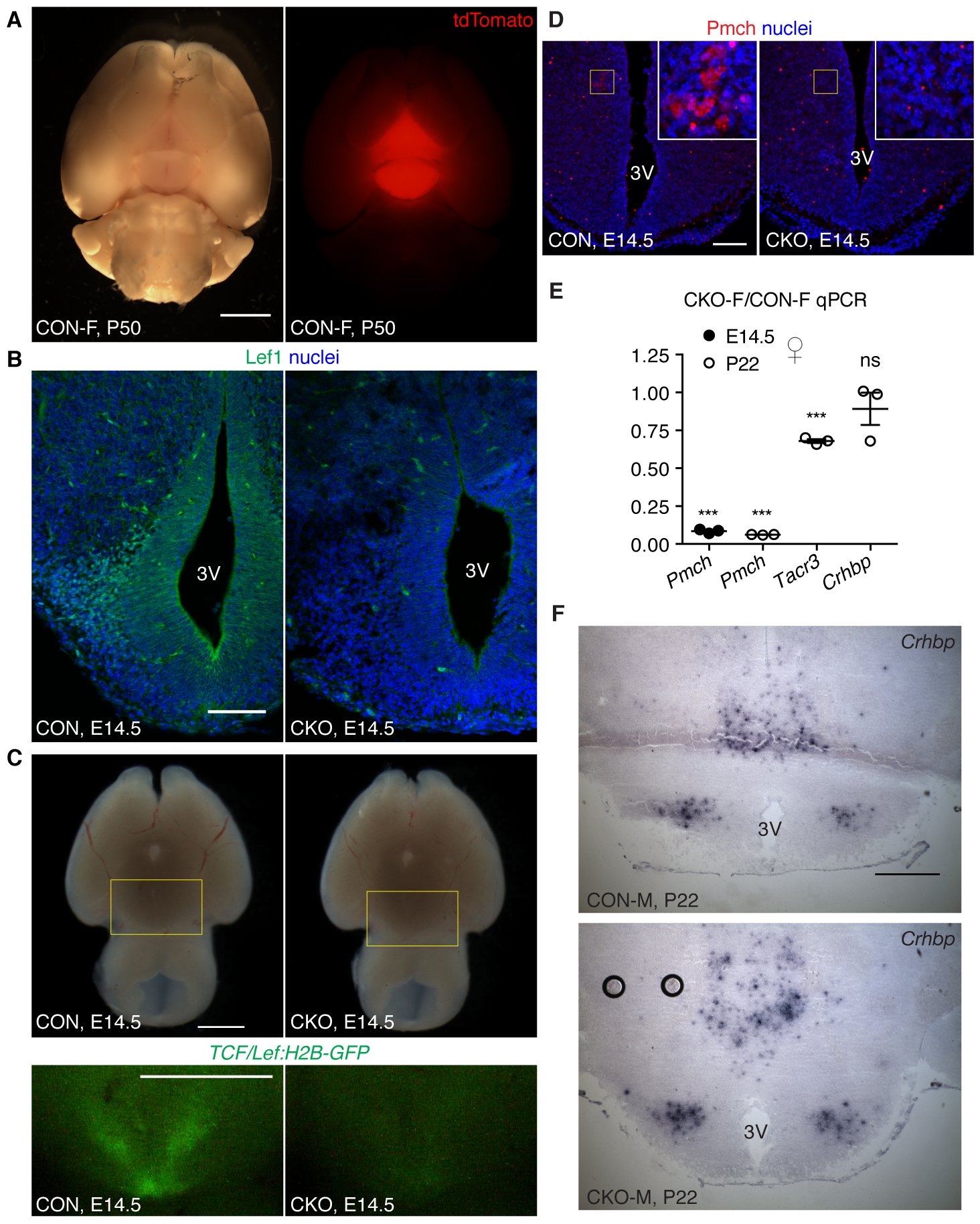

Supplement: S5 Fig — (A) P50 female Nkx2-1Cre/+;Lef1flox/+;RosatdTomato/+ (CON-F) expresses tdTomato in the hypothalamus. Bright field (left) and red fluorescence (right) ventral view images of the same brain with anterior on top are shown. Representative images are shown for at least 3 adult brains dissected. (B) Immunostaining for Lef1 in the hypothalamus of E14.5 Lef1CON (CON) and Lef1CKO (CKO). Coronal images are z-projections of 16 μm confocal optical sections, shown with dorsal side on top. Representative images are shown for at least 2 replicates tested. (C) Immunostaining for Wnt reporter TCF/Lef:H2B-GFP. Hypothalamic green fluorescence (below) views of yellow rectangles in bright field (above) view images of the same brain are shown, respectively. Images are whole mount ventral views with anterior side on top, acquired with the same setting for CON and CKO. Representative images are shown for at least 3 replicates tested. (D) Immunostaining for Pmch in the E14.5 hypothalamus. Higher magnification views of yellow rectangles are shown in the insets. Coronal images are z-projections of 16 μm confocal optical slices, shown with dorsal side on top. (E) qPCR analysis for female shows hypothalamic gene expression in E14.5 and P22 CKO-F relative to CON-F. Data are mean ± SEM. ***P < 0.001, ns. P > 0.05 by unpaired Student t tests. (F) Twenty-five μm coronal section in situ hybridization for Crhbp in the male P22 ventral premammillary and posterior hypothalamus, shown with dorsal side on top. Representative images are shown for at least 2 replicates tested. 3V: third ventricle. All scale bars are 100 μm except 500 μm in (F). Raw data can be found in S1 Data. (TIF) [file pbio.2002257.s005.tif]

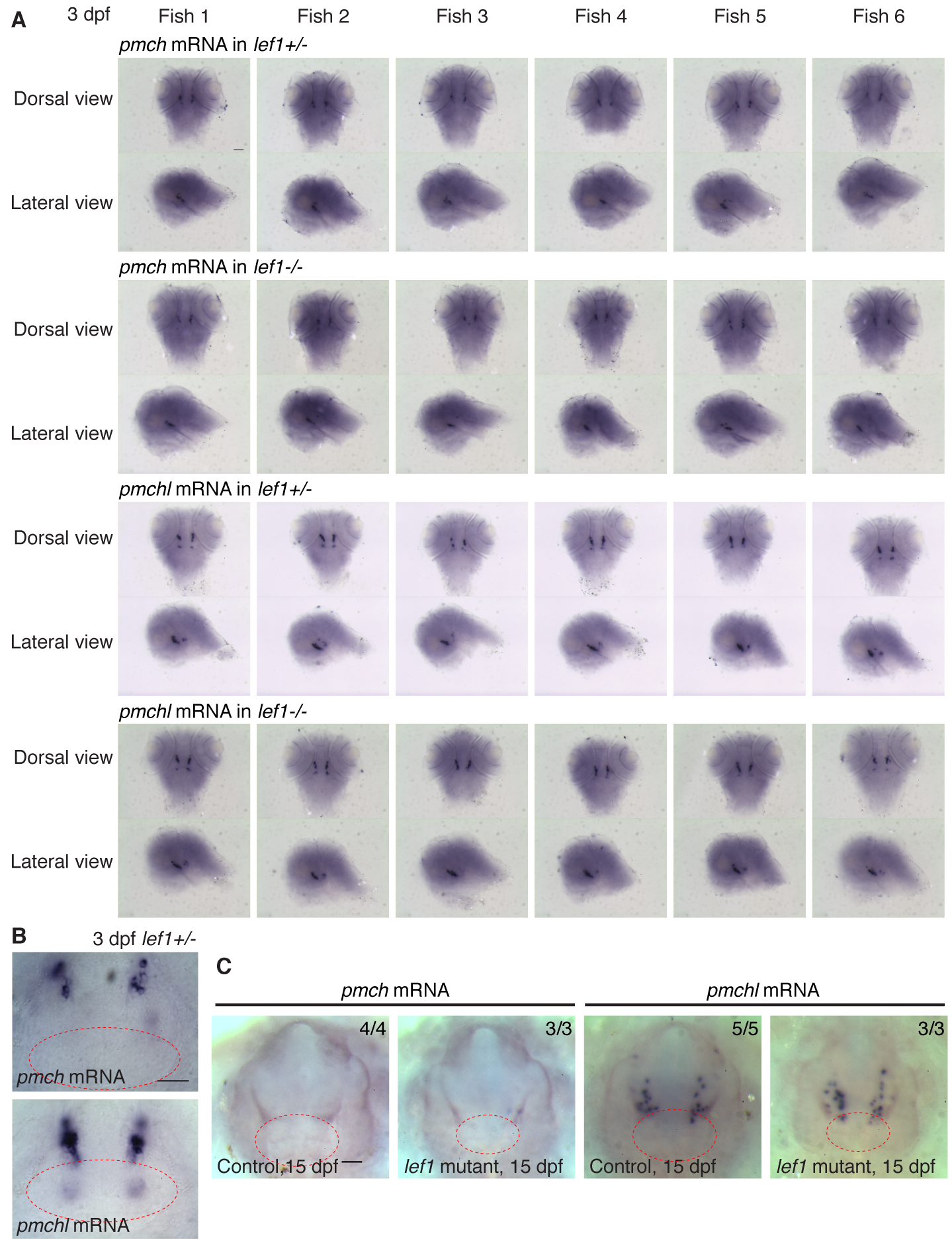

Supplement: S6 Fig — (A-C) Whole mount in situ hybridization images for pmch and pmchl (pmch, like) in the hypothalamus of 3 dpf (A and B) and 15 dpf (C) zebrafish control and lef1 mutant embryos. Images of dorsal views (anterior on top) and lateral views (dorsal on top and anterior on the left) of the same individual lef1+/- or lef1-/- fish were shown in (A). Representative ventral view images of 3 dpf lef1+/- (B), 15 dpf control and lef1 mutant (C) brains centered on the caudal hypothalamus (dashed red outlines) with anterior on top. Number of fish with representative gene expression among total number of fish is labeled on the right upper corner of each image in (C). Scale bar: 100 μm in (A and C); 5 μm in (B). (TIF) [file pbio.2002257.s006.tif]

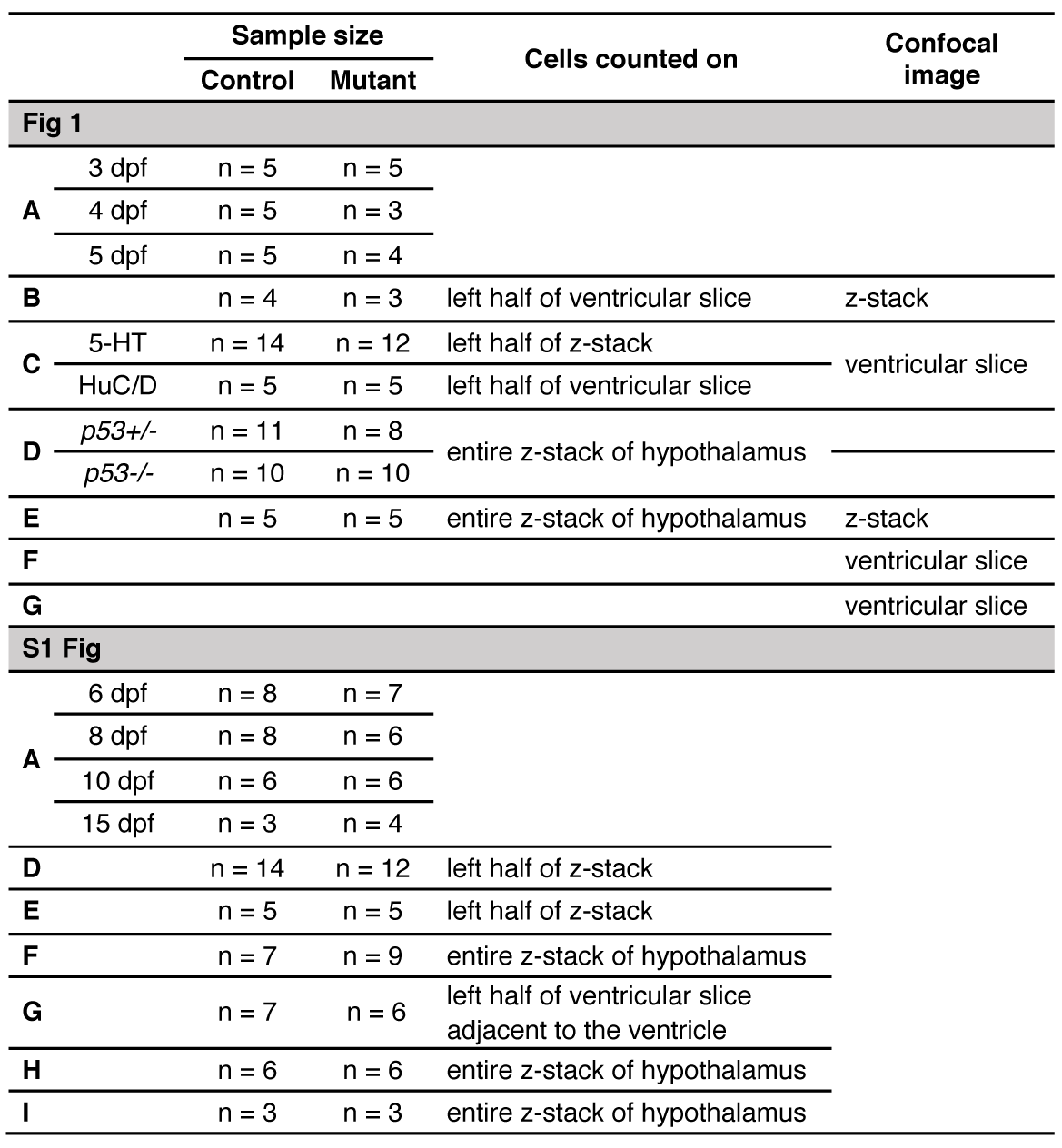

Supplement: S1 Table — (TIF) [file pbio.2002257.s007.tif]
